# Ligation-assisted homologous recombination enables precise genome editing by deploying both MMEJ and HDR

**DOI:** 10.1093/nar/gkac118

**Published:** 2022-02-25

**Authors:** Zhihan Zhao, Peng Shang, Fanny Sage, Niels Geijsen

**Affiliations:** Dept. of Anatomy & Embryology, Leiden University Medical Center, Einthovenweg 20, 2300 RC Leiden, The Netherlands; Dept. of Anatomy & Embryology, Leiden University Medical Center, Einthovenweg 20, 2300 RC Leiden, The Netherlands; Dept. of Anatomy & Embryology, Leiden University Medical Center, Einthovenweg 20, 2300 RC Leiden, The Netherlands; Dept. of Anatomy & Embryology, Leiden University Medical Center, Einthovenweg 20, 2300 RC Leiden, The Netherlands

## Abstract

CRISPR/Cas12a is a single effector nuclease that, like CRISPR/Cas9, has been harnessed for genome editing based on its ability to generate targeted DNA double strand breaks (DSBs). Unlike the blunt-ended DSB generated by Cas9, Cas12a generates sticky-ended DSB that could potentially aid precise genome editing, but this unique feature has thus far been underutilized. In the current study, we found that a short double-stranded DNA (dsDNA) repair template containing a sticky end that matched one of the Cas12a-generated DSB ends and a homologous arm sharing homology with the genomic region adjacent to the other end of the DSB enabled precise repair of the DSB and introduced a desired nucleotide substitution. We termed this strategy ‘Ligation-Assisted Homologous Recombination’ (LAHR). Compared to the single-stranded oligo deoxyribonucleotide (ssODN)-mediated homology directed repair (HDR), LAHR yields relatively high editing efficiency as demonstrated for both a reporter gene and endogenous genes. We found that both HDR and microhomology-mediated end joining (MMEJ) mechanisms are involved in the LAHR process. Our LAHR genome editing strategy, extends the repertoire of genome editing technologies and provides a broader understanding of the type and role of DNA repair mechanisms involved in genome editing.

## Graphical Abstract

**Graphical Abstract:**
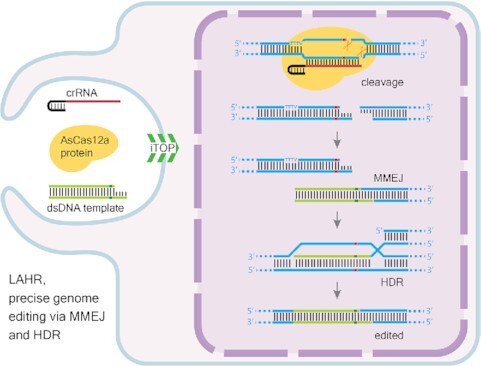
Ligation-assisted homologous recombination enables precise genome editing by deploying both MMEJ and HDR.

## INTRODUCTION

Over the past decade, genome editing technologies have become one of the essential molecular techniques in biomedical research (1–4). Given that many human diseases have a genetic basis, these genome editing technologies, especially precise insertion, deletion, or replacement of parts of the genome, hold tremendous promise for the treatment of monogenetic disorders (5–8). Since their discovery, CRISPR/Cas systems have quickly become the editing technology of choice for targeted genome manipulation (9–12). CRISPR/Cas systems employ a short RNA molecule, the guide RNA, to lead a CRISPR effector nuclease to the genomic target position of interest, creating a double strand break (DSB) at the genomic target site. Subsequently, DNA damage-induced endogenous DNA repair machineries are recruited towards the cut site and repair the damage, by which the genomic manipulations occur and the ‘editing’ is finalized (13,14).

Cas12a (previously known as Cpf1), like Cas9, is a single-effector CRISPR protein (15,16). Cas12a differs from Cas9 with respect to several important properties (17). Cas12a naturally employs a single CRISPR RNA (crRNA) as guide RNA, which is substantially shorter than the engineered single guide RNA (sgRNA) used for Cas9 (18). In addition, Cas12a recognizes a T-rich protospacer adjacent motif (PAM) sequence, comparing to the G-rich PAM recognized by Cas9 (15). And importantly, Cas12a uses a single RuvC catalytic domain to cleave both the target and non-target strands, creating 5′-overhang sticky ends (19,20), while Cas9 employs two nuclease domains RuvC and HNH generating a blunt-ended DSB at the target locus (21,22). Due to these properties, Cas12a greatly expands the genome editing toolbox (23).

The ability to generate sticky ends at the target DNA cleavage site makes Cas12a a useful tool for *in vitro* DNA assembly (24,25). Recently, Li *et al.* developed a new Cas12a-based genome editing method named MITI (microhomology-dependent targeted integration) (26), which utilized the Cas12a-generated compatible sticky ends between transgene and target site termini to direct a site-specific gene insertion. The authors demonstrated how MITI could be applied to insert a gene of interest together with a positive selection cassette into a single Cas12a target-site in the genome. Yet, the need for a selection cassette and the reported inaccurate integration of the targeting construct at the 5′ and 3′ junctions restrained its application, especially in the context of gene therapeutics.

We initially set out with a similar goal to explore the utility of two AsCas12a cleavage sites on the genome to excise the target sequence, and then replace it with a double-stranded DNA (dsDNA) insert containing compatible sticky ends. We called this strategy ‘Cut-And-Paste Repair’ (CAPR) and observed that CAPR was able to rescue the EGFP fluorescent signal in the EGFP*^Δ^^fluor^* reporter cells, yet the repair efficiency turned out to be rather low. Unexpectedly, we noticed that one of our controls, the repair insert of CAPR with only a single AsCas12 cleavage (single cut control), rescued the EGFP fluorescence with much higher efficiency. We further polished this unexpected finding by using a repair template containing a short dsDNA homologous arm plus a single sticky end fitting an AsCas12a-generated compatible end on the genome, to deliver a desired nucleotide substitution located on the homologues arm. We named this strategy ‘Ligation-Assisted Homologous Recombination’ or LAHR. Using a reporter cell line, we demonstrated that LAHR was able to effectively repair the point mutation thereby restoring EGFP fluorescence. LAHR has a relatively high repair efficiency both in a reporter cell line and when editing endogenous genes. Finally, we explored the DNA repair mechanism(s) underlying LAHR using RNAi-mediated knockdown of different DNA DSB repair pathways. We demonstrated that LAHR utilizes a combination of both MMEJ (in a resection-independent manner) and HDR pathways. We believe that LAHR opens new possibilities for precise genome editing, complementary to Cas-nuclease-induced HDR using ssODN template.

## MATERIALS AND METHODS

### Reagent sharing

Recombinant SpCas9 and AsCas12a proteins (Supplementary Table S11), as well as single-copy EGFP*^Δ^^fluor^* and EGFP*^Y66S^* reporter HAP1 cell lines and Beta-2-microglobulin (B2M)-deficient HAP1 cell line (HAP1*^B2M–/^^–^*) are available through Divvly (https://divvly.com/geijsenlab).

### Cell lines and cell culture

HAP1 cells derived from the KBM-7 cell line were a main cell line used in this study (27). All the reporter cell lines based on HAP1 cells were cultured in Iscove's modified Dulbecco's medium (IMDM) (Gibco), supplemented with 10% fetal bovine serum and 1% penicillin/streptomycin; HEK293 cells were cultured in Dulbecco's modified Eagle's medium (DMEM) (Gibco), with 10% fetal bovine serum and 1% penicillin/streptomycin; C2C12 cells were cultured in DMEM, with 15% fetal bovine serum and 1% penicillin/streptomycin; ARPE19 cells were cultured in DMEM/F12 (Gibco), with 20% fetal bovine serum, 56 mM sodium bicarbonate and 2 mM l-glutamine. All cells were grown at 37°C in a humidified atmosphere containing 5% CO_2_.

### Molecular cloning

Two targeting constructs, pAAVS1-EGFP*^Δ^^fluor^* and pAAVS1-EGFP*^Y66S^*, were made to generate the single-copy EGFP*^Δ^^fluor^* and EGFP*^Y66S^* reporter HAP1 cell lines (Supplementary figure S1). We used previously published plasmid ‘CRISPR-SP-Cas9 Reporter’ (Addgene #62733) as backbone (27). The reporter EGFP mutants, EGFP*^Δ^^fluor^* and EGFP*^Y66S^*, were synthesized as gBlock gene fragments (IDT, Supplementary Table S2), and amplified by PCR using the primer pair: Fw 5′-ATGGTGAGCAAGGGC GAGG-3′, Rv 5′-TTACTTGTACAGCTCGTCCATGCC-3′; The homologous arms were amplified by PCR from genomic DNA of the host HAP1 cells, with the primer pairs: Fw 5′-GCTCAGTCTGGTCTATCTGCC-3′ and Rv 5′-TGTCCCTAGTGGCCCCAC-3′ for the left homologous arm (1011 bp); Fw 5′-GGATTGGTGACAGAAAAGCCC-3′ and Rv 5′-TCCCCTGCTTCTTGGCC-3′ for the right homologous arm (1107 bp). The minimal ubiquitous chromatin opening element (UCOE) fragment was amplified by PCR from the plasmid pMH0001 (Addgene #85969) (28), with the primer pair: Fw 5′-ATCGAATTCGGGAGGTGGTCC-3′, Rv 5′-AGGACTCCGCGCCTACAG-3′. The EGFP mutants were cloned into the backbone plasmid between NotI and BamHI sites. The left homologous arm and the minimal UCOE fragment were cloned into SpeI site upstream of the human EF1alpha promoter. A polyA sequence (79 bp) and the right homologous arm were cloned downstream of puromycin resistance gene using ClaI site.

The expression plasmid pET15B_AsCas12a was constructed using a previously published SpCas9 expression plasmid ‘Sp-Cas9’ (Addgene #62731) as backbone (27). Briefly, *Escherichia coli*. codon optimized AsCas12a coding sequence (including NLS and 6x His tag at C-terminal) was synthesized by GenScript (GenScript). The AsCas12a coding sequence then was amplified by PCR with the primer pair: Fw 5′-AGGAGATATACCATGACCCAGTTTG-3′, Rv 5′-GTTAGCAGCCGGATCCTTAATG-3′, and cloned into the backbone plasmid between NcoI and BamHI sites.

All the restriction enzymes used were from New England Biolabs (NEB). All PCR-amplified fragments were cloned into backbone plasmids with In-Fusion HD Cloning Plus kit (Takara).

### Generation of single-copy EGFP mutants reporter cell lines

To generate the single-copy EGFP*^Δ^^fluor^* and EGFP*^Y66S^* reporter cell lines, we targeted the reporter genes into the genome of HAP1 cells at the human *AAVS1* locus that located in the first intron of human *PPP1R12C* gene (28). To enhance the efficiency of HDR, we co-transfected the donor plasmid together with recombinant SpCas9 protein and AAVS1-T2 guide RNA (10) into HAP1 cells by using Lonza Nucleofection system following the manufacturer's protocol. In brief, 4 μg of donor plasmid, 150 pmol of recombinant SpCa9 protein (75 μM) and 300 pmol of Alt-R 2-part guide RNA (100 μM) (IDT) were added into 100 μl of Lonza Nucleofection buffer for cell line (Lonza). 1 × 10^6^ HAP1 cells were resuspended with the complete Lonza Nucleofection buffer, and the nucleofection was performed in a Lonza Nucleofector 2b device with the program of ‘Cell-line T-030’. 24 hours after transfection, the addition of puromycin (1:20 000) was applied in the cell culture to start the positive selection. The concentration of puromycin was doubled after 3 days. After 10 days of positive selection, survival cells were single-cell sorted onto a 96-well plate. We typically sorted 96 single cells for each targeting. The correctly targeted HAP1 clones were verified by border-PCR (Supplementary figure S1). All primers were listed in (Supplementary Table S1)

### Expression and purification of recombinant AsCas12a protein

To express and purify the recombinant AsCas12a protein, we adapted a previously published method (27). In brief, the expression plasmid pET15B_AsCas12a was introduced into the One Shot BL21(DE3) chemically competent *E coli*. cells (Invitrogen) that was priorly transformed with a chaperone plasmid pG-Tf2 (Takara). A single colony was grown overnight in 50 ml LB medium pre-culture containing 150 μg/ml ampicillin, 34 μg/ml chloramphenicol and 1% glucose, at 37°C, with shaking at 225 rpm. 10 ml pre-culture was then added into 400 ml of LB medium (150 μg/ml ampicillin, 34 μg/ml chloramphenicol, 1% glucose, 5 ng/ml tetracycline, and 2.5 mM MgCl_2_) and cultured at 37°C, with shaking at 225 rpm until OD reached 0.5. After IPTG was added to a final concentration of 1 mM, the culture was incubated overnight at 25°C with shaking at 225 rpm. Harvested cells were lysed in the lysis buffer (50 mM NaH_2_PO_4_, 1 M NaCl, 1 mM MgCl_2_, 0.2 mM PMSF, 10 mM beta-2-mercaptoethanol and 0.1 mg/ml lysozyme, pH 8.0, supplemented with cOmplete Protease Inhibitor Cocktail Tablets, 1 tablet/50 ml and Benzonase Nuclease, 25 U/ml) with sonication at 4°C. The sonicated cell lysate was solubilized with the NDSB buffer (50 mM NaH_2_PO_4_, 1 M NaCl, 2 M NDSB-201, 2.5 mM MgCl_2_ and 10 mM beta-2-mercaptoethanol, pH 8.0) at 4°C with rotation. The solubilized cell lysate was cleared by centrifugation at 10 000 ×*g* for 60 min at 4°C. The Ni^2+^ affinity column chromatography was performed using a 5-ml HisTrap™ HP column with an ÄKTA pure 25 FPLC system (GE Healthcare). AsCas12a protein was eluted in the elution buffer (50 mM NaH_2_PO_4_, 1 M NaCl, 500 mM GABA, 500 mM imidazole, 2.5 mM MgCl_2_ and 5 mM beta-2-mercaptoethanol, pH 8.0) with a continuous concentration gradient. The target elution peak was buffer exchanged into the protein storage buffer (25 mM NaH_2_PO_4_, 500 mM NaCl, 250 mM GABA, 150 mM glycerol, 75 mM glycine, 1.25 mM MgCl_2_, 2 mM beta-2-mercaptoethanol, pH 8.0), using a HiLoad 26/600 Superdex 200 pg gel filtration column (GE Healthcare). The purified AsCas12a protein then was concentrated to 75 μM using Amicon Ultracel Centrifugal Filters (MWCO 100 kDa) (Millipore). All chemicals and reagents were purchased from Sigma (Sigma).

### Guide RNAs and repair donors used in CAPR and LAHR

Guide RNAs used in this study are synthetic guide RNA produced by IDT (IDT). Sequences of guide RNAs are listed in Supplementary Table S3. Lyophilized guide RNA was dissolved in nuclease-free water to reach a final concentration of 75 μM. The dsDNA repair inserts (used in CAPR), or templates (used in LAHR) were generated by annealing two reverse complement ssDNA oligos. All ssDNA oligos were synthesized by IDT (IDT), and the sequences are listed in Supplementary Tables S4–S7. Lyophilized ssDNA oligos were dissolved in the oligo annealing buffer (30 mM HEPES, pH 7.5; 100 mM potassium acetate) to reach a concentration of 100 μM. A pair of oligos for annealing was mixed in equal volume, heated at 95°C for 5 min and cooled down to room temperature.

### Induced transduction by osmocytosis and propanebetaine (iTOP)

The recombinant SpCas9 or AsCas12a proteins, guide RNAs and repair donors were simultaneously transduced into target cells by using the iTOP method we described previously (27). One day prior to transduction, the reporter cells were plated in the Matrigel-coated wells on 96-well plates at 30–40% confluence, such that on the day of transduction, cells were at 70–80% confluence. Next day, for each well of the 96-well plate, 50 μl of iTOP mixture that contains 20 μl of transduction supplement (Opti-MEM media supplemented with 542 mM NaCl, 333 mM GABA, 1.67× N2, 1.67× B27, 1.67× non-essential amino acids, 3.3 mM Glutamine, 167 ng/ml FGF2 and 84 ng/ml EGF), 10 μl of CRISPR nuclease protein (75 μM), 10 μl of guide RNA (75 μM) and the excess volume of nuclease-free water to reach a 50-μl total volume, were prepared. For the no-protein control, 10 μl of protein storage buffer was used instead of SpCas9 or AsCas12a protein; and for the no-guide control, the equal volume of nuclease-free water was used to replace the guide RNA. After the culture medium was removed, the iTOP mixture was added onto the cells. The plate then was incubated in a cell culture incubator for 45 min, after which the iTOP mixture was gently removed and exchanged for 200 μl of regular culture medium (pre-warmed to 37°C).

### Electroporation

To deliver the LAHR components into reporter cells by electroporation, we used a Lonza Nucleofector system that includes Cell Line Nucleofector Kit V and Nucleofector 2b Device (Lonza), following the manufacturing protocol. In brief, 1 × 10^6^ target cells were re-suspended in 100 μl of supplemented Nucleofector solution V buffer that contains AsCas12a RNP together with repair templates (50–500 pmol of each component in the molar ratio of 1:1 were used in experiments). The electroporation was performed with the program ‘Cell-line T-020’ in the Nucleofector 2b Device. After electroporation, the cells were incubated at 37°C and the culture medium was changed after 16 h.

### FACS analysis

To verify the gene editing efficacies in the single-copy EGFP*^Δ^^fluor^* and EGFP*^Y66S^* reporter HAP1 cell lines, FACS analyses were performed 48 h after iTOP transduction. Cells in each well were trypsinized and resuspended in 200 μl of FACS buffer (5% FBS in 1 × DPBS) containing 1:1000 DAPI (4′,6-diamidino-2-phenylindole) DNA dye (Sigma). For the beta-2-microglobulin (B2M)-deficient HAP1 cells, 48 h after iTOP transduction, cells from each well were firstly trypsinized and then incubated in 50 μl of staining solution (1% FITC-conjugated anti-human HLA-A, B, C antibody (Biolegend) in FACS buffer) for 10 min at 4°C. After washing three times with 1× DPBS, cells were resuspended in 150 μl FACS buffer containing 1:1000 DAPI DNA dye. FACS analyses were carried out on a CytoFLEX LX system (Beckman). In all experiments, the total number of 10 000 events were acquired and were gated based on side and forward light-scatter parameters. Constitutive EGFP-expressing control HAP1 cells were used to adjust the parameters for the identification and gating of EGFP/FITC positive cells. The EGFP/FITC signal was detected using the 488 nm diode laser for excitation and the 525/40 nm filter for emission.

### Cell viability assay

Post-iTOP cell viability was analyzed using an MTS Assay Kit (Abcam) following the manufacturer's instructions. In Brief, cells were seeded on a 96-well plate at 30–40% of confluence, iTOP transduction was performed when the confluence reached 70–80%. 12–24 h after the iTOP transduction, 5 μg/ml of 3-(4,5-dimethylthiazol-2-yl)-5-(3-carboxymethoxyphenyl)-2-(4-sulfophenyl)-2H-tetrazolium (MTS reagent) was added into each well and incubated at 37°C for 90 min. The absorbance was measured on a BIO-RAD XMark Microplate spectrophotometer at 490 nm (BIO-RAD).

### RNA interference

To perform the small interfering RNA (siRNA)-mediated knockdown for the selected genes that are involved in different DNA DSB repair pathways, the EGFP*^Y66S^* reporter HAP1 cells were plated on 48-well plates and transfected with 3 pmol of each targeting siRNA (Supplementary Table S8) using Lipofectamine RNAiMAX Transfection Reagent following the manufacturer's protocol (Thermo Fisher). All siRNA oligos in this study were ordered from Thermo Fisher (Thermo Fisher).

### Total RNA isolation and cDNA synthesis

Six days after siRNA transfection, total RNA was extracted from transfected cell samples and siRNA-free control transfection samples with TRIzol™ Reagent (Sigma), following the manufacturer's protocol. The precipitated RNA pellets were dissolved in 20 μl nuclease-free water (Invitrogen). Next, the total RNA samples were treated with RQ1 RNase-free DNase (Promega), following the manufacturer's protocol. cDNA was synthesized using SuperScript III Reverse Transcription (RT) Kit (Invitrogen), following the manufacturer's protocol. Briefly, 5 μg of DNase-treated total RNA, 1 μl of random primers (50 ng/μl), 1 μl of 10 mM dNTPs and nuclease-free water were added up to reach a total volume of 13 μl. The mixture was heated at 65°C for 5 minutes and snap-cooled on ice for 1 min. On top of the 13-μl mixture, 4 μl of 5× First-Strand Buffer, 1 μl of 0.1 M DTT, 1 μl of RNaseOUT and 1 μl of SuperScript III RT (200 units/μl) were added. Following an incubation at 25°C for 5 min, the cDNA synthesis reaction was performed by incubating at 50°C for 1 h. The RT reaction was inactivated by heating at 70°C for 15 minutes.

### Quantitative analysis of RNAi knockdown by qPCR

Expression levels of the target genes were quantified by qPCR on siRNA-treated samples and respective siRNA-free control samples. All the cDNA samples were from 3 times of independent siRNA transfection experiments (3× biological replicates). For each cDNA sample, 3 qPCR reactions (3× technical replicates) were setup for the target gene and for the *GAPDH* internal control respectively. Each qPCR reaction contains 5 μl of 2× iQ™ SYBR Green Supermix (BIO-RAD), 1 μl of forward-reverse primer mix (10 μM), 1 μl of cDNA template and 3 μl of Nuclease-free water in a total volume of 10 μl. qPCR reactions were run on a BIO-RAD CFX96 Real-Time system (BIO-RAD). The amplification program was initiated at 95 °C for 3 minutes, followed by 40 cycles of 95°C for 10 s, 60°C for 20 s. After amplification, an additional thermal denaturizing cycle (temperature ranged between 65°C and 95°C in 0.5°C increments) was performed to obtain the melting curves of the qPCR products and to verify amplification specificity. Expression level was calculated by subtracting internal control (*GAPDH*) quantification cycle (Cq) value from the Cq value of the target gene to normalize for total input, resulting in a ΔCq value. Relative expression level was calculated as 2^−ΔCq^. All the gene specific primers for qPCR reaction were listed in Supplementary Table S8.

### T7 endonuclease I assay

To assess the AsCas12a cleavage efficiencies of the target sites in the EGFP*^Δ^^fluor^* mutant. We applied T7 Endonuclease I (T7E1) assay following the AsCas12a targeting cleavage conducted by iTOP. Three days after iTOP AsCas12 RNP transduction, cells were harvested to isolate genomic DNA using DNeasy Blood & Tissue Kit (Qiagen). Primers used for genomic DNA amplification are listed in Supplementary Table S9. The gel-purified PCR products were then subjected to T7E1 assay with Alt-R Genome Editing Detection Kit (IDT) following the manufacturing protocol. Briefly, in a thermocycler, 500 ng of purified PCR product were denatured at 95°C for 5 min and re-annealed at −2°C per second temperature ramp to 85°C, followed by a −0.1°C per second ramp to 25°C, and cooled to 4°C. The rehybridized PCR product was incubated with 3 U T7E1 enzyme at 37°C for 30 min. The enzyme-treated products were resolved on a 2% agarose gel. Densitometry analysis was performed with ImageJ (29).

### Next-generation sequencing

Amplicon sequencing with Illumina MiSeq platform was performed as previously described (30). In brief, the amplicon libraries were built following a two-round PCR protocol. The first round of PCR (PCR 1) amplified the target genomic loci by using locus-specific primer pairs tailed with Illumina sequencing adapters (Supplementary Table S10). PCR 1 was performed using a Q5 High-Fidelity PCR Kit (NEB), following the manufacturing protocol. Each PCR 1 reaction (50 μl) contained 50 ng of genomic DNA template, 0.5 μM of each primer, 200 μM of dNTP, 0.02 U/μl of Q5 High-Fidelity DNA polymerase and 1x Q5 reaction buffer. The PCR 1 amplification initiated with a denaturation step at 98°C for 2 min, followed by 30 cycles of denaturation at 98°C for 10 s, primer annealing at 61°C for 30 s, and primer extension at 72°C for 30 s. Upon completion of the cycling steps, a final extension at 72°C for 5 min was done and then the reaction was held at 12°C. The gel-purified PCR 1 products were then used as the templates of the second round PCR (PCR 2) where the PCR 1 products were indexed by the amplification using unique illumine barcoding primers. PCR 2 was as well performed with the Q5 High-Fidelity PCR Kit, in a 25-μl setup using 10 ng of purified PCR 1 product as template in each reaction. For the PCR 2 amplification, a denaturation step initiated at 98°C for 12 s, followed by 12 cycles of denaturation at 98°C for 10 s, primer annealing at 61°C for 30 s, and primer extension at 72°C for 30 s. When the final extension at 72°C for 5 min was done the reaction was held at 12°C. Next, gel-purified PCR 2 products (pooled amplicons) were sequenced on an Illumina MiSeq platform, by which we generated about 30 000 total reads for each experimental sample. Sequencing reads were demultiplexed using MiSeq Reporter (Illumina). Alignment of amplicon sequences to a reference sequence was performed using CRISPResso2 (31). The editing efficiency was calculated as: the percentage of [the number of reads of edited]/[the number of total reads].

## RESULTS

### Cut-And-Paste Repair (CAPR) utilizing sticky ends generated by AsCas12a cleavage

**Figure 1.**
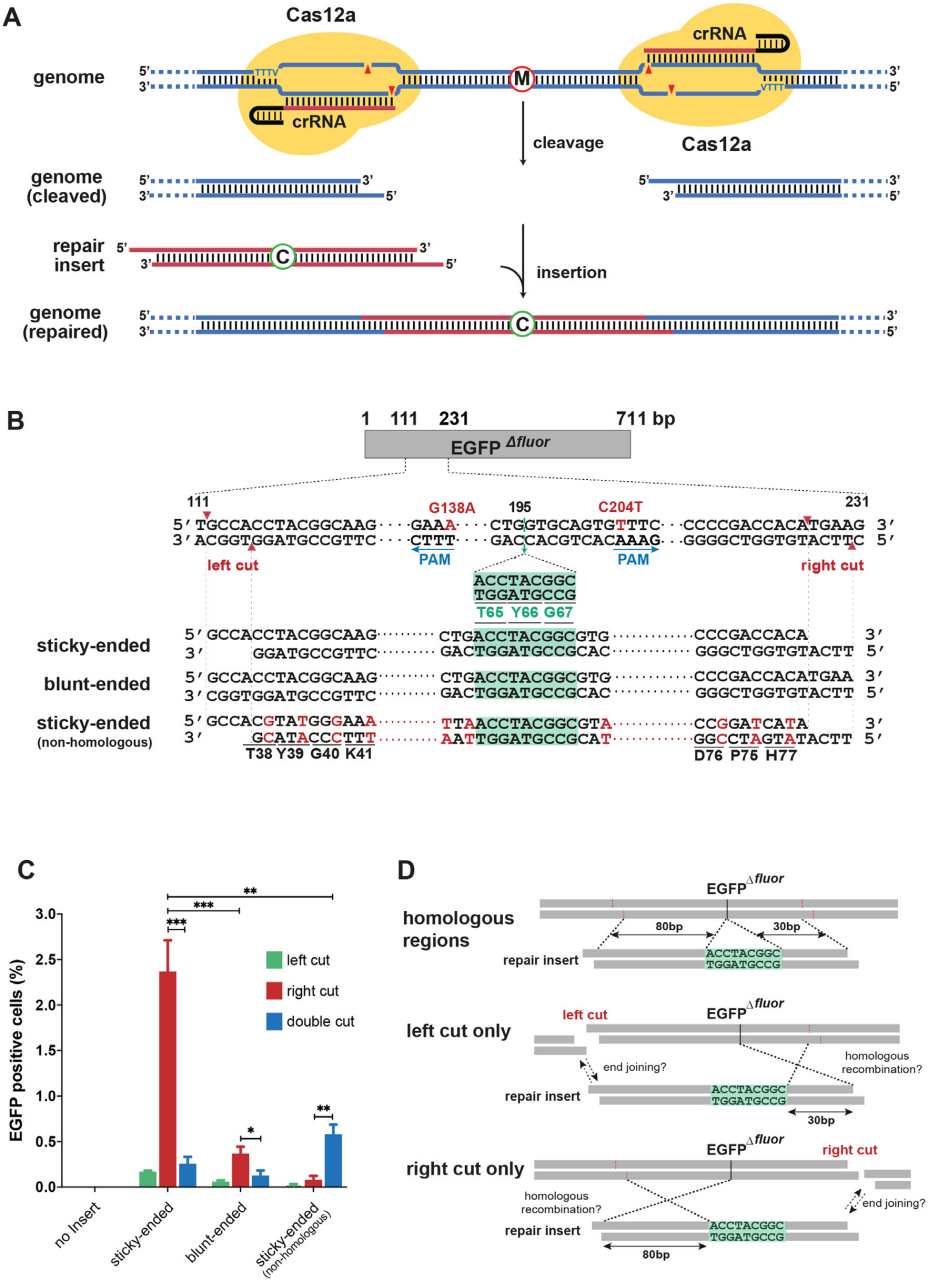
Conducting precise genome editing by CAPR. (**A**) Schematic representation of the CAPR strategy, in which two molecules of Cas12a protein were guided by two crRNAs to target and excise a genomic region containing a malicious mutation (red-circled M); the cleavage created two staggered ends; a given repair insert possessing two compatible sticky ends and a correction (blue-circled C) ligated to the compatible ends on the genome to perform the repair. (**B**) Schematic representation of the EGFP*^Δ^^fluor^* reporter. The AsCas12a editing regions between T111 and G231 were shown in a double-stranded format, in which silent mutations G138A and C204T (red uppercases) were introduced to generate two AsCas12a PAM sites (underlined by blue arrows, and the arrows show the direction of the PAM sequences). Two AsCas12a cut sites: ‘left cut’ and ‘right cut’ are indicated by red arrowheads. The fluorophore-coding sequence (encoding T65, Y66 and G67) removed behind G195 is highlighted in green. Three repair inserts, ‘sticky-ended’, ‘blunt-ended’ and ‘sticky-ended (non-homologous)’ containing the fluorophore-coding sequence (green-highlighted) are indicated below. The ‘sticky-ended (non-homologous)’ insert contained multiple silent mutations (red uppercases) resulting in loss of homology at the DNA level, whilst preserving the amino acid sequence (corresponding amino acids are shown underneath) (**C**) The EGFP*^Δ^^fluor^* mutant was repaired by different repair inserts in different cleavage scenarios. The repair efficiency is indicated by the percentage of EGFP positive cells (Flow cytometry analysis). Error bars indicate the standard deviation of the average of n = 3 parallel samples. The experiment was repeated three times and a representative dataset was presented here. Statistical test: two-tailed unpaired *t*-test, * *P* < 0.05, ** *P* < 0.01, *** *P* < 0.001. (**D**) A hypothetic model of the repair in the context of the Cas12a cut. The repair insert contained two homologous regions to the EGFP*^Δ^^fluor^* reporter gene, 80 bp and 30 bp respectively, flanking the EGFP fluorophore-coding sequence (green highlighted). In the case of ‘left cut only’, the effective homologous arm of the repair insert was 30 bp, while the cut only at the right side, the effective homologous arm was 80 bp.

As shown in Figure 1A, our initial strategy was to try and take advantage of 5′ overhangs introduced by AsCas12a to ligate a dsDNA fragment possessing the complementary ends (repair insert) in a ‘cut-and-paste’ fashion (Figure 1A). We referred to this strategy as ‘Cut-And-Paste Repair’ (CAPR), and it formed the basis of our initial design and subsequent refinements, as described below. For our experimental setup, we built a cell-line carrying a single-copy, fluorescence-impaired EGFP reporter (EGFP*^Δ^^fluor^*) (Supplementary figure S1), in which the codons encoding threonine (T) 65, tyrosine (Y) 66 and glycine (G) 67, were deleted to abrogate the EGFP fluorescence. Furthermore, the EGFP*^Δ^^fluor^* construct harbored two additional silent mutations, G138A and C204T, to introduce two AsCas12a PAM sites (TTTV) flanking the deletion site (Figure 1B). The resulting EGFP*^Δ^^fluor^* cell-line now allowed AsCas12a-mediated targeting and removal of a 115-bp region containing the *Δfluor* deletion, exposing two sticky ends that were compatible with a simultaneously transduced repair insert. Correct ligation of the repair insert would restore EGFP fluorescence and could be quantified by FACS analysis.

We previously reported how a combination of small molecules could trigger the efficient uptake and intracellular release of recombinant protein and small oligonucleotides, a method termed iTOP (27). We employed iTOP to simultaneously deliver all the components of CAPR (recombinant AsCas12a protein, crRNA pair, and the repair insert) into the EGFP*^Δ^^fluor^* reporter cells. The repair efficiency was quantified by FACS analysis and Sanger sequencing 48 hours after the iTOP transduction. We observed that CAPR enabled the replacement of mutated region between two cut sites and rescued the EGFP fluorescence, yet at rather low efficiency (<0.5%) (Figure 1C). Unexpectedly, in the negative controls in which only a single left- or right-side cut was made, EGFP fluorescence was restored as well, and the one with the single right-side cut resulted in a more than 9-fold higher repair efficiency compared to CAPR (Figure 1C). A similar trend was observed in the control group using a blunt-ended insert (Figure 1C). Given that the sequences of both the sticky-ended and the blunt-ended repair inserts were homologous to the corresponding regions in the target reporter gene (Figure 1D), we were intrigued by the possibility that a single sticky overhang was sufficient to trigger effective repair, potentially by combining sticky-ended ligation with HDR of the remaining template. To test if HDR was involved in the repair process, we applied a non-homologous insert together with the single-cut controls and, as expected, observed no fluorescence rescue (Figure 1C). Nonetheless, when this non-homologous repair insert was used in combination with both crRNAs, this resulted in rescue of fluorescence, demonstrating that our non-homologous repair insert can repair the target sequence by the CAPR mechanism (Figure 1C, Supplementary Figure S2A). While both blunt and sticky-ended repair inserts could rescue the fluorescence in the single cut scenario, the blunt-ended insert yielded a lower repair efficiency since it did not match the 5′ overhang created by AsCas12a, whereas the insert with a matching sticky end favored the repair efficiency (Figure 1C). An explanation for the observation that the ‘right cut only’ exhibited higher repair efficiency than the ‘left cut only’ condition, could be that in the ‘right cut only’ situation (Figure 1D), the homologous arm of the repair insert (80 bp) is much longer compared to the ‘left cut only’ situation (30 bp). An alternative explanation could be however, a difference in AsCas12a cutting efficiencies at the left and right cut sites. To exclude this possibility, we determined the indel frequencies at both cut sites after AsCas12a cleavage (Supplementary Figure S2B). We observed that the AsCas12a cutting efficiencies on these two sites were similar, excluding the possibility that the observed repair differences were caused by differences in AsCas12a cleavage activity at these sites.

Taken together, our data suggested that a single sticky end generated by AsCas12a cleavage was able to ligate to the compatible end of the repair insert by end-joining mechanisms, thereby allowing the homologous region of the insert to recombine to the corresponding region on the genome by a homology-directed process (Figure 1D). The mechanism and factors that impact this possible ‘Ligation-assisted Homologous Recombination’ (LAHR), was explored further, as outlined below.

### The sticky end and the homologous arm are indispensable for the LAHR template

**Figure 2.**
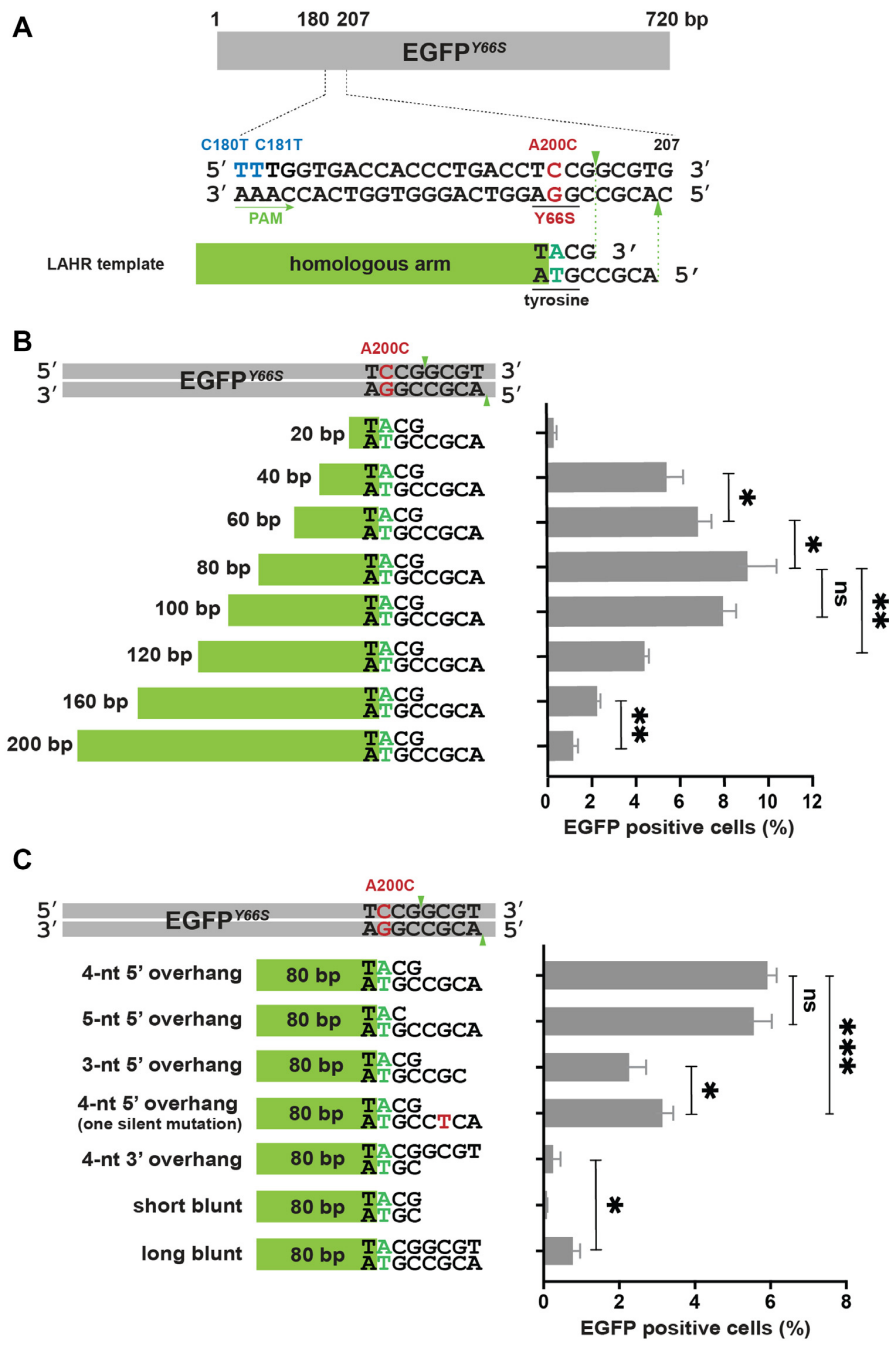
LAHR utilizes both the homologous arm and the sticky end of the repair template. (**A**) Schematic of the EGFP*^Y66S^*reporter sequence and the repair template. The dsDNA region between T180 and G207 indicates the AsCas12a editing region, in which the silent mutations, C180T and C181T (blue uppercases) were introduced to generate an AsCas12 PAM sequence (underlined by a green arrow, and the arrow orientated the direction of the PAM sequence); the cleavage site is indicated by green arrowheads; the missense mutation A200C (red uppercase) causes a tyrosine-to-serine substitution (Y66S) that eliminated the EGFP fluorescence. The repair template contained a sticky end, which was compatible to the AsCas12a-generated distal sticky end on the reporter gene, and a homologous arm (green box). Adjacent to the sticky end of the repair template a repairing A/T base pair (green uppercases) was introduced to restore the codon of tyrosine. (**B**) Correction of the A200C mutation using repair templates with a same sticky end, but varying lengths of the homologous arms (from 20 bp to 200 bp). Indicated percentage of EGFP-positive cells was determined by flow cytometry (See also supplementary figure S12). Error bars indicate the standard deviation of the average of n = 3 parallel samples. The experiment was repeated three times and a representative dataset is presented here. Statistical test: two-tailed unpaired t-test, ns *P* > 0.05, * *P* < 0.05, ** *P* < 0.01. (**C**) Correction of the A200C mutation using repair templates sharing a same 80-bp homologous arm, but with indicated sticky ends. Indicated percentage of EGFP-positive cells was determined by flow cytometry (See also supplementary figure S12). Error bars indicate the standard deviation of the average of *n* = 3 parallel samples. The experiment was repeated three times and a representative dataset is presented here. Statistical test: two-tailed unpaired *t*-test, ns *P* > 0.05, * *P* < 0.05, *** *P* < 0.001.

To explore the LAHR hypothesis further, we built another single copy EGFP mutant reporter, EGFP*^Y66S^*, cell line (Supplementary Figure S1), in which a missense mutation A200C converted the EGFP tyrosine (Y) 66 into a serine (S), thereby eliminating the EGFP fluorescence (32). The EGFP*^Y66S^* gene construct also featured two additional silent mutations, C180T and C181T, to introduce a single AsCas12a PAM site just upstream of the A200C mutation (Figure 2A). To repair the A200C mutation and restore EGFP fluorescence, LAHR templates were designed to contain the following elements (Figure 2A, ‘LAHR template’): (i) a sticky end that matches the PAM-distal sticky end of the AsCas12a-generated DSB ends on the target reporter gene, (ii) an A/T base pair located on the homologous arm that can repair the A200C mutation (Figure 2A, green ‘A/T’ pair) and (iii) a homologous arm that shared the homology with the corresponding region adjacent to the PAM-proximal end of the AsCas12a-generated DSB (Figure 2A, green rectangular).

To evaluate the role of the length of the homologous arm in the LAHR process, we designed a series of eight LAHR templates sharing the same sticky end, and with different lengths of the homologous arms, varying from 20 to 200 bp (Figure 2B). The LAHR template together with the AsCas12a RNP were transduced into the EGFP*^Y66S^* reporter cells by iTOP, followed by FACS analysis to quantify gene editing efficiency. We observed that the length of homologous arm was an important determinant of LAHR efficiency, and that 80 bp represented an optimal length in this case (Figure 2B). Shorter homologous arms, especially the 20-bp one showed low repair efficiency, suggesting that a short homologous arm was ineffective in driving the homologous recombination-mediated integration of the distal end of the LAHR template. On the other hand, templates with lengths over 120 bp showed decreased repair efficiencies possibly due to a decreased ability to diffuse into the nucleus.

Next, we explored whether the presence of a compatible sticky end on the LAHR template was required in the LAHR process. Since the optimal length of the homologous arm has been determined to be 80 bp (Figure 2B), we generated LAHR templates with the same 80-bp homologous arm and varied the 3′ terminal ends (Figure 2C). We observed that a template with a 4-nt 5′ overhang that perfectly matched the PAM-distal sticky end generated by AsCas12a resulted in the highest repair efficiency. LAHR templates with 4-nt or 5-nt 5′ overhangs demonstrated similar repair efficiencies, which reflected the ability of AsCas12a to cleave the non-target DNA strand at either the 18th or the 19th base behind the PAM sequence, yielding a 5-nt or a 4-nt 5′ overhang respectively (15). The templates with a 3-nt 5′ overhang or with a single nucleotide-mismatched 4-nt 5′ overhang (introducing a silent mutation) could still repair the mutation albeit with lower efficiency. In contrast, blunt-ended or 3′-overhang sticky-ended LAHR templates that did not match the AsCas12a-generated sticky end at all exhibited extremely low repair efficiencies (Figure 2C). Taken together, our results indicated that both an appropriate homologous arm and a compatible sticky end were required to achieve LAHR.

### Characterization of LAHR

Since the iTOP transduction technology allowed simultaneous delivery of AsCas12a protein, crRNA, and LAHR template, we examined how the quantitative ratio of these components to affect LAHR efficiency. We had previously noticed that editing efficiencies plateaued when the concentration of SpCas9 protein reached 15–20 μM (not shown). We observed that the amount of AsCas12a protein used in LAHR exhibited similar plateau effect when the concentration was reaching 15 μM (Supplementary figure S3A). Next, we titrated the crRNA, at a Cas12a concentration of 15 μM. As shown in Supplementary figure S3B, the optimal molar ratio between AsCas12a protein and crRNA was 1:4. With the optimal AsCas12a protein-crRNA ratio, we made a titration curve of the LAHR template (Supplementary figure S3C). As shown LAHR editing efficiency was linearly correlated with the concentration of LAHR template in the transduction mixture, suggesting that the concentration of the repair template at the Cas12a target site was the rate-limiting step in LAHR-based repair. We did not observe differences in cell viability in all the test conditions (Supplementary figure S4), excluding the possibility that our results were influenced by differences in cell viability under these different conditions.

**Figure 3.**
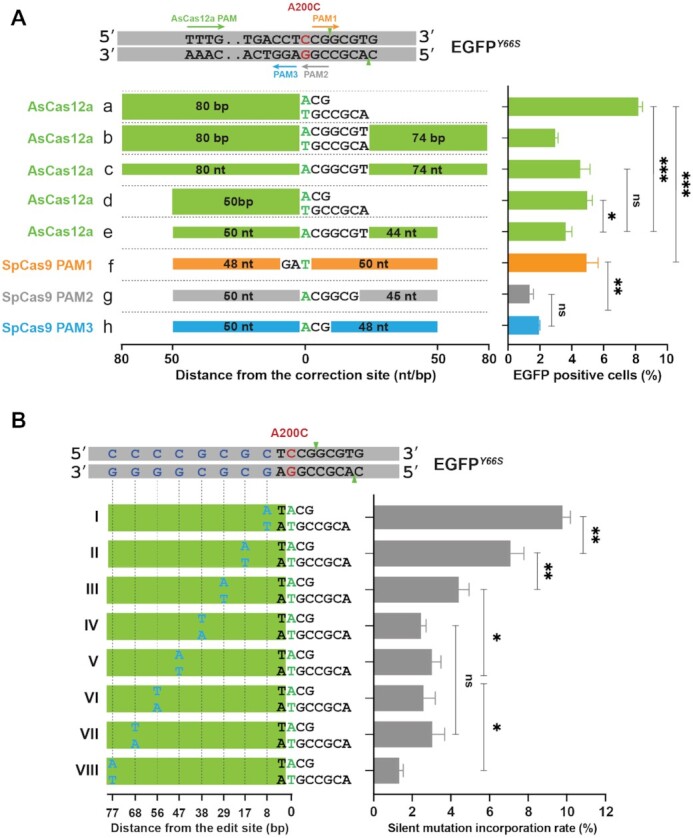
Characterization of LAHR. (**A**) Comparison between LAHR and HDR in repair of the EGFP*^Y66S^*reporter. Cells were targeted using AsCas12a RNP and different repair templates as indicated, (a) a LAHR template with an 80-bp homologous arm and a compatible sticky end; (b) a 160-bp dsDNA template; (c) a 160-nt ssODN template; (d) a LAHR template with a 50-bp homologous arm; (e) a 100-nt ssODN template. For the SpCas9 targeting, the following repair templates were used: (f) a 100-nt ssODN template for PAM1; (g) a 100-nt ssODN template for PAM2; (h) a 100-nt ssODN template for PAM3. Templates (g) and (h) were the same sequence, and the template (f) was their reverse complement sequence. For all repair templates from (a) to (f), the homologous arms are presented as coloured boxes, and different colours indicated different PAM usages. The numbers in the boxes indicate the size of the homologous arm. The correction base (A or T) or base pair (A/T) was in green uppercases. The scale beneath repair templates indicated the distance between each end and the correction site. Indicated percentage of EGFP-positive cells was determined by flow cytometry (See also supplementary figure S12). Error bars correspond to the standard deviation of the average of n = 3 parallel samples. The experiment was repeated three times and a representative dataset is presented here. Statistical test: two-tailed unpaired t-test, ns *P* > 0.05, * *P* < 0.05, ** *P* < 0.01, *** *P* < 0.001. (**B**) The repair templates (I–VIII) contained the same A/T base pair (green uppercase) to correct the A200C mutation. A single base pair change (blue uppercases), introducing a silent mutation, on each template was distributed along the homologous arm (green box). The scale at bottom indicates the distance between the silent-mutation-inducing substitution and the repairing A/T base pair. Shown is the percentage incorporation of each silent mutation determined by NGS analysis. Error bars indicate the standard deviation of the average of *n* = 3 parallel samples. Statistical test: two-tailed unpaired *t-*test, ns *P* > 0.05, * *P* < 0.05.

Next, we compared LAHR efficiency with simple HDR in our single-copy EGFP*^Y66S^* reporter cells. As shown in Figure 3A, in the context of AsCas12a-induced DSB, the efficiency of LAHR using a template with an 80-bp homologous arm was significantly higher than the simple HDR using a 160-nt ssODN template with two 80bp homologous arms. Commonly used ssODN repair templates for HDR are 90–100 nt in length (33), so we also did a similar comparison between LAHR using a template with a 50-bp homologous arm and HDR using a 100-nt ssODN template. Here too, LAHR exhibited higher repair efficiency. As we also noticed that HDR using a 100-nt or 160-nt ssODN template did not show significantly different repair efficiencies (Figure 3A), thus we used 100-nt ssODN repair templates for HDR in following experiments. In addition to comparing LAHR and HDR efficiencies at the same AsCas12a-created DSB, we also compared the efficiencies between LAHR and the SpCas9-mediated HDR in repair of the EGFP*^Y66S^* mutation. In line with recent reports (34,35), we also found AsCas12a exhibited a preference for an ssODN of the non-target sequence, while SpCas9 preferred an ssODN of the target strand sequence (Supplementary figure S5). Near the A200C mutation (Figure 3A, indicated as a red C/G pair), three SpCas9 PAM sites are available to conduct Cas9-mediated HDR (Figure 3A). We would like to note that, in the current experimental setup, the condition of Cas9-mediated HDR was not fully optimized, and we can therefore not conclude that LAHR editing efficiency is higher than Cas9-mediated HDR. However, our data suggests that LAHR editing efficiency is at least comparable to Cas9-mediated HDR. In scenarios where Cas9-mediated HDR fails to achieve adequate repair efficiencies, or at loci where Cas9 PAM sites are not available, LAHR could therefore be a practical alternative approach to achieve precise gene repair.

The LAHR template featured a rather short single-sided homologous arm carrying an intended nucleotide substitution, we wondered how the location of the nucleotide substitution could influence the LAHR efficiency. To address this question, we designed another experiment based on the same single-copy EGFP*^Y66S^* reporter cell line used above, in which we performed LAHR with a series of repair templates carrying not only the nucleotide substitution to repair the A200C mutation, but also an additional silent mutation distributed along the homologous arm on each LAHR template (Figure 3B). The incorporation rate of each silent mutation would indicate its position effect. The incorporation rates of silent mutations were determined by NGS analysis. We observed that incorporation rate of silent mutations diminished the more these were located toward the blunt end of the LAHR template (Figure 3B), which demonstrates that, as expected, a mutation is more likely to be introduced by LAHR if it is closer to the Cas12a cut site. Interestingly, when we analyzed corresponding EGFP fluorescence restoration rates by FACS, we found that silent-mutations in the middle of the homologous arm demonstrated diminished EGFP restoration efficiencies (Supplementary figure S6, templates I - IV) compared to mutation located near the Cas12a cut site or near the blunt end of the LAHR template (Supplementary figure S6, templates V - VIII). We also performed NGS analysis to investigate the actual repair rate of the A200C mutation corresponding to each FACS sample, which corroborated the FACS results (Supplementary figure S6). These data suggested that mutations in the middle of the LAHR template impair efficient HDR-mediated integration resulting in lower EGFP restoration rates.

Previous reports have demonstrated that mutation disrupting the PAM sequence or seed region can avoid ‘re-cutting’ of the edited genome and increase editing efficiency (36,37). We designed a LAHR targeting strategy to test whether introduction of silent mutations disrupting the Cas12a PAM site and/or seed region on the LAHR template could similarly enhance LAHR targeting efficiency (Supplementary figure S7A). The result demonstrated that disruption of either PAM sequence or seed region significantly enhances LAHR editing efficiency (Supplementary figure S7B), but unexpectedly, this effect was lost when both PAM and seed sequences were disrupted (Supplementary figure S7B). Similar results were observed when ssODN templates were used (Supplementary figure S7A and B). Possibly, mutations in both the PAM and seed sequences create multiple mismatches, disrupting the homology between the LAHR template and the target genome, which may counteract the benefits gained from avoiding ‘re-cutting’.

In addition to using iTOP to deliver the LAHR components, we assessed the applicability of LAHR with a non-iTOP delivery method. With the Lonza Nucleofection system, we applied LAHR to repair the same mutation in EGFP*^Y66S^* reporter cell line. We observed that nucleofection-mediated delivery of AsCas12a RNP and LAHR template similarly allows LAHR-mediated restoration of EGFP expression (Supplementary figure S8).

### LAHR-mediated precise genome editing targeting endogenous genes

**Figure 4.**
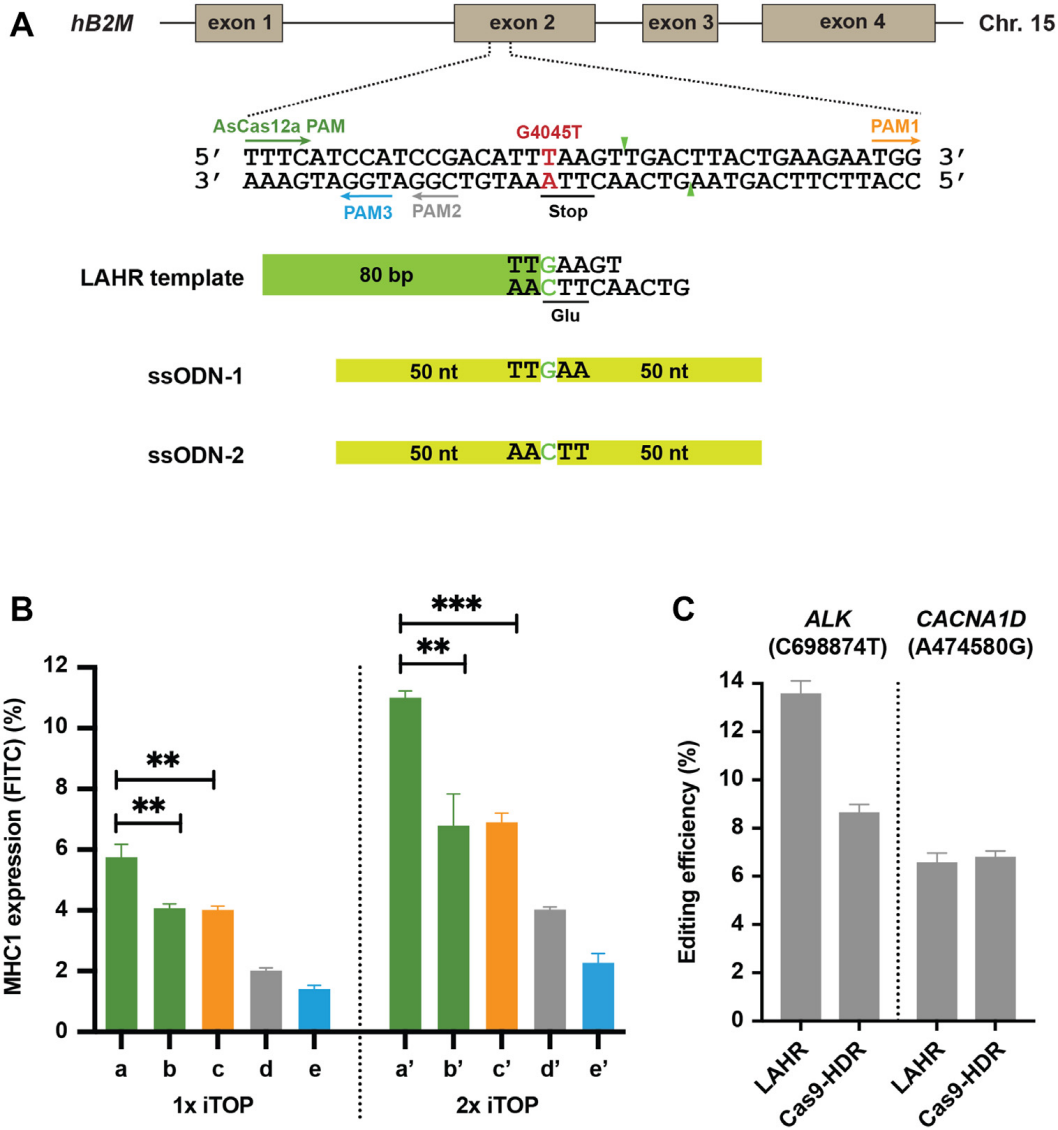
Introducing single nucleotide substitutions in endogenous genes by LAHR or HDR. (**A**) Schematic depiction of the human Beta-2-microglobulin gene with a nonsense mutation G4045T (in red text) that introduces a premature stop codon (underlined). One AsCas12 PAM (green arrow) and three SpCas9 PAMs (orange, grey and blue arrows) flank the G4045T mutation. The AsCas12a cleavage site is indicated by green arrowheads. In addition to the LAHR template, two 100-nt ssODN templates, ssODN-1 (from the positive strand) and ssODN-2 (from the negative strand) were used to repair the DSBs induced by AsCas12a or SpCas9 using the different PAMs. (**B**) The bar chart shows the repair efficiencies of different nucleases, PAM sites and repair template combinations as indicated. Repair efficiency was measured as percentage FITC-positive cells in flow cytometry analysis of MHC1 surface expression. The colours of bars reflect different PAM sites consistent with the colours of PAMs indicated in (A) (Green AsCas12a PAM; Orange SpCas9 PAM1; Grey SpCas9 PAM2; Blue SpCas9 PAM3). Bar a and a’ indicate LAHR-mediated repair; b and b’ indicate AsCas12a-mediated HDR using the template ssODN-1; c and c’ indicate the SpCas9-mediated HDR (PAM1) using the template ssODN-2; d and d’ indicate the SpCas9-mediated HDR (PAM2) using the template ssODN-1; e and e’ indicate SpCas9-mediated HDR (PAM3) using the template ssODN-1. For the bar a-e, one-round iTOP transduction was performed, while the bar a’-e’ exhibit the repair efficiencies from two rounds of iTOP transductions. Indicated percentage of FITC-positive cells was determined by flow cytometry (See also supplementary Figure S12). Error bars correspond to the standard deviation of the average of *n* = 3 parallel samples. The experiment was repeated three times and a representative dataset is presented here. Statistical test: two-tailed unpaired *t*-test, ** *P* < 0.01, *** *P* < 0.001. (**C**) Two single nucleotide substitutions, C698874T and A474580G, were respectively introduced in human *ALK* and *CACNA1D* genes (Supplementary Figure S9) by LAHR or by Cas9-mediated HDR. The bar chart shows a comparison of the editing efficiencies between LAHR and Cas9-mediated HDR. Error bars correspond to the standard deviation of the average of *n* = 3 independent biological replicates.

Our proof-of-concept data and characterization of LAHR in the reporter cell-line demonstrated that LAHR could efficiently repair a point-mutation in an EGFP*^Y66S^* reporter system. We next compared LAHR gene editing efficiency with AsCas12a or SpCas9-mediated HDR in endogenous genes. Previously, we had introduced a homozygous nonsense mutation G4045T (Glu55-STOP) in exon 2 of the human beta-2-microglobulin (*B2M*) gene resulting in a *B2M* knockout phenotype (HAP1*^B2M–/–^*, unpublished). In the absence of B2M protein, the MHC1 complex cannot be presented at the cell surface. In this system, restoration of surface MHC1 expression can be used to quantify the restoration of B2M expression. There is an AsCas12a PAM sequence, TTTC, 14-bp upstream of this G4045T mutation, and the AsCas12a cleavage site is 4 bp downstream from the G4045T mutation (Figure 4A). There are also three SpCas9 PAM sites surrounding the G4045T mutation, allowing to repair the mutation by Cas9-mediated HDR as well (Figure 4A, indicated in orange, grey and blue). We transduced recombinant AsCas12a protein, the crRNA, and the LAHR template into the HAP1*^B2M–/–^* cells by iTOP. As a control, a 100-nt ssODN template was applied to perform the HDR induced by the same AsCas12a cleavage. As shown in Figure 4B, the repair efficiency of LAHR was higher than that of the AsCas12a-mediated HDR using an ssODN template (Figure 4B, a, b and a’, b’), consistent with the result of the comparative analysis performed in the EGFP*^Y66S^* reporter cells. We also compared LAHR to SpCas9-mediated HDR utilizing the three available SpCas9 PAMs with the corresponding 100-nt ssODN templates (Figure 4B, c, d, e, and c’, d’, e’). In the repair of this mutation, LAHR performed better than the Cas9-mediated HDR (Figure 4B), which once more demonstrated that LAHR could deliver precise genome editing at loci where the Cas9-mediated HDR may be inefficient. Moreover, we also noticed that by applying a second round of LAHR, the end repair efficiency of LAHR was almost doubled (Figure 4B).

In addition to repairing a targeted nonsense mutation in the endogenous B2M gene, we also assessed the ability of LAHR to precisely introduce single nucleotide substitutions in other endogenous genes. In a previously published report by Wang *et al.* (34), point mutations, C698874T and A474580G (Supplementary Figure S9), were introduced into the human *ALK* and *CACNA1D* genes respectively, by either AsCas12a or SpCas9-mediated HDR using ssODN templates. Here, we introduced the same substitutions by LAHR, and compared the efficiency of LAHR to the SpCas9-mediated HDR (Supplementary Figure S9). For the substitution C698874T in *ALK*, LAHR exhibited above 30% higher editing efficiency compared to Cas9-mediated HDR, while for A474580G in *CACNA1D*, the editing efficiencies from these two methods were similar (Figure 4C).

### Mechanisms underlying LAHR

**Figure 5.**
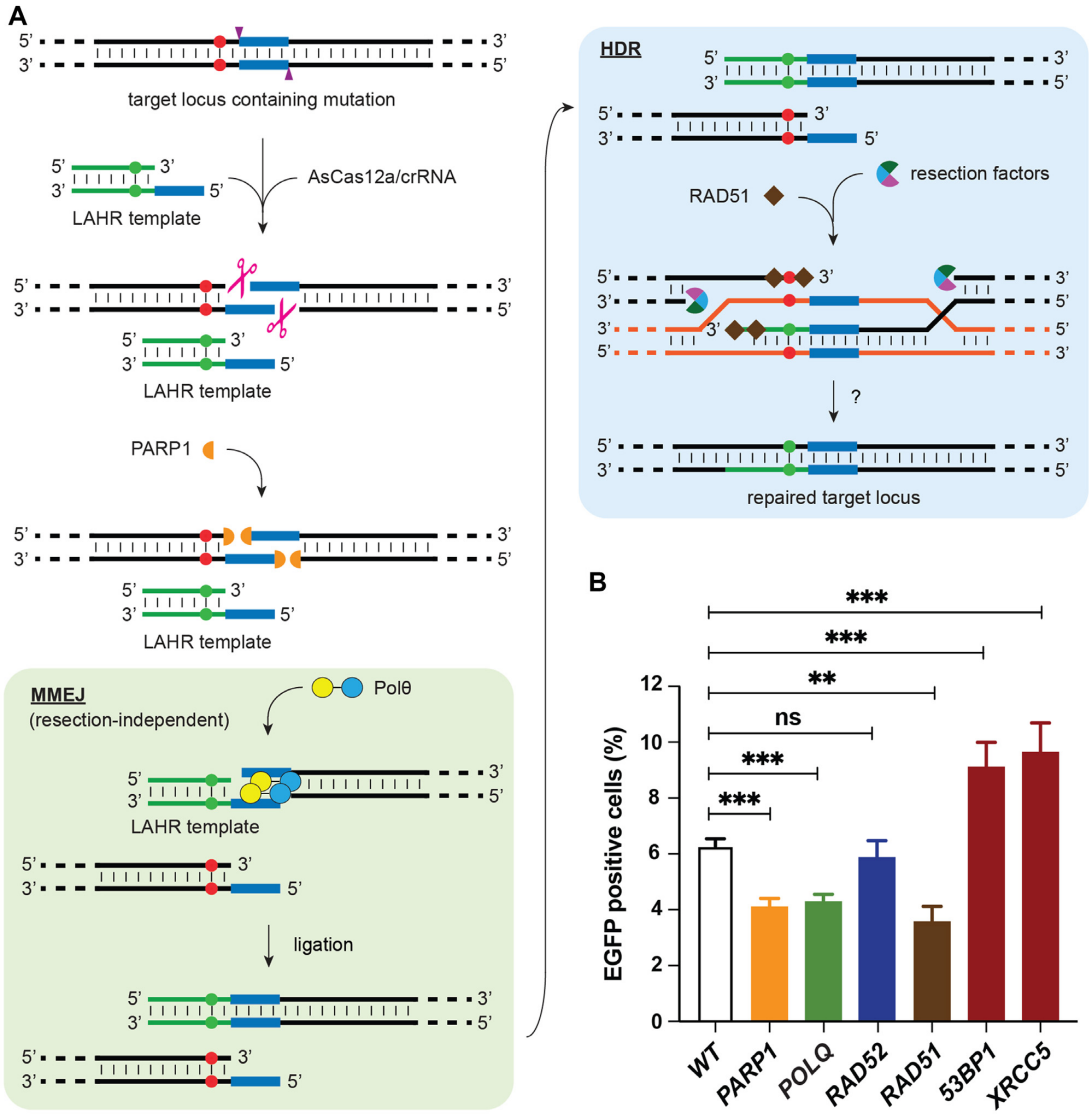
DNA repair mechanisms underlying LAHR. (**A**) Schematic representation of LAHR. AsCas12a/crRNA-RNP-mediated cleavage of a target sequence containing a mutation (red dots on both strands). The AsCas12a cut site is indicated by purple arrowheads on both strands. The AsCas12a-generated DSB yields two staggered ends with 5′ homologous overhangs (blue blocks). PARP1 accumulation at the DSB ends recruits downstream factors involved in different DNA DSB repair pathways. MMEJ-mediated repair is highlighted in a light green box, where the recruited Polθ pairs two compatible 5′ homologous overhangs located on the downstream DSB end and on the LAHR template respectively. The MMEJ process here does not involve resection. After ligation of the LAHR template to the downstream DSB end, by the resection-independent MMEJ, HDR (highlighted in a light blue box) is employed to incorporate the correct base (green dots on the LAHR template) into genome. The orange dsDNA template indicates the sister chromatid. The question mark at the last step of HDR indicates a potential mismatch repair or base-excision repair procedure involved to convert the mutation to a correct base. (**B**) Effect of siRNA knockdown of key DNA repair pathways on LAHR. Selected gene targets were knocked down by siRNA targeting. The bar graph shows the repair efficiencies of EGFP*^Y66S^* cells under different gene knockdown conditions, as percentages of EGFP positive cells determined by flow cytometry analysis. (Supplementary Figure S12). Error bars correspond to the standard deviation of the average of *n* = 3 parallel samples. The experiment was repeated three times and a representative dataset is presented here. Statistical test: two-tailed unpaired *t*-test, ns *P* > 0.05, ** *P* < 0.01, *** *P* < 0.001.

In the LAHR process, the AsCas12a-generated DSB is repaired by using a repair template featuring a sticky end (5′ overhang) and a short double-stranded homologous arm. Since both features are indispensable to accomplish the repair, we therefore assumed that there might be two distinct DSB repair pathways involved in the LAHR process. We hereby hypothesized that LAHR could utilize the 5′ homologous overhangs to ligate the repair template to the AsCas12a-created compatible DSB end via an MMEJ pathway and is subsequently completed by a homology-directed integration of the homologous arm (Fig 5A). To verify this hypothesis, we examined the effect of small interfering RNA (siRNA) knockdown of select genes involved in DSB-repair on LAHR efficiency. Specifically, we performed knockdown of: (i) *PARP1*, which is an upstream gene involved in DSB detection and recruitment of downstream DSB repair machineries (38); (ii) *POLQ* encoding the DNA polymerase Polθ, which plays a pivotal role in microhomology identification and annealing in MMEJ (39); (iii) *RAD52*, which is essential in single-strand annealing (SSA) process where RAD52 binds the 3′ overhangs created by resection to facilitate end recognition and pairing (40,41); (iv) *RAD51* is an essential gene in HDR, which binds to resection-created 3′ single strand and leads the strand to invade template DNA based on homologies (42); (v) *53BP1* and 6) *XRCC5* (encoding KU80) are key genes involved in the NHEJ pathway, both of which inhibit the resection process which is essential for MMEJ, SSA and HDR (43). The efficacy of the siRNA knockdown of these factors was confirmed by qPCR (Supplementary Figure S10). Upon siRNA knockdown of the indicated pathways in EGFP*^Y66S^* reporter cells, we determined LAHR efficiency by FACS analysis of EGFP expression (Figure 5B).

As expected, *PARP1* knockdown decreased EGFP*^Y66S^* repair efficiency, as the upstream inhibition DSB detection and repair machinery recruitment could fundamentally restrain all DSB repair. Knockdown of Polθ, essential for MMEJ, also significantly decreased the repair efficiency, which indicated that Polθ-mediated MMEJ plays an important role in the LAHR process. After MMEJ, the ligated repair template could potentially be utilized through SSA, a process coordinated by RAD52. However, *RAD52* knockdown did not affect LAHR efficiency, suggesting that LAHR does not involve RAD52-dependent SSA repair. Knockdown of *RAD51* resulted in a significantly decreased repair efficiency, which indicated that besides MMEJ, HDR likely was another essential pathway employed in LAHR. In addition, we also observed that knockdown of either *53BP1* or *XRCC5*, consistently resulted in a slight but significant increase in LAHR-mediated repair, in line with the role of these factors in determining the balance between NHEJ and other resection-dependent repair pathways.

Canonical MMEJ is initiated by strand resection, which creates 3′ overhangs to expose matched microhomologies (44). In LAHR, the AsCas12a-created genomic 5′ overhang and the compatible 5′ overhangs on the repair template seem to bypass the need for resection. To further examine the possibility of a resection-independent MMEJ mechanism employed in LAHR, we designed a LAHR template containing a 4-nt 3′ homologous overhang, that could be utilized in MMEJ only after resection exposing the matching homology on the genome (Supplementary figure S11A). The repair efficiency using the 4-nt 3′ overhang template was significantly lower than the repair achieved by using a regular 5′ overhang LAHR template (Supplementary figure S11B), indicating that the MMEJ mechanism employed in LAHR favors a pre-existing 5′ homologous overhang via a resection-independent pathway.

Altogether, these results clearly verified our hypothesis that both HDR and MMEJ pathways were essential for LAHR-mediated gene repair, and the MMEJ in LAHR takes place in a resection-independent manner.

## DISCUSSION

In the current study we describe a novel method for precise genome editing using an AsCas12a-generated DSB, and a dsDNA repair template containing a matching 5′ overhang and a short double-stranded homologous arm. We call this method LAHR, for ‘Ligation-Assisted Homologous Recombination’. LAHR is the first precise genome editing tool that deploys both HDR and MMEJ mechanisms to repair an AsCas12a-generated DSB and introduced a desired nucleotide substitution. The complementary 5′ overhangs created by AsCas12a at the target site in the genome lock the repair template in place and ligate via a resection-independent MMEJ pathway, while template integration is completed by HDR. As summarized in Figure 5, these two processes elegantly work together. The AsCas12a-cleaved genomic DSB end and repair template both contain homologous 5′ overhangs, such that they enter the MMEJ pathway at the level of Polθ, skipping the need of initial strand resection. The remaining homologous arm of the template recombines by HDR with an unbroken sister chromatid. Finally, the base mismatch created by the template mutation is resolved, potentially by base-excision repair or mismatch repair.

In Cas12a-medaited genome editing, a LAHR template is more efficient than an ssODN template in introducing a specific mutation. The comparison between LAHR and SpCas9-mediated HDR (using ssODN templates) is difficult, if not impossible, due to differences in PAM sites, cut sites, and repair template preference between Cas12a and Cas9 gene editing systems. Yet our data demonstrates that, at different loci examined, LAHR repair efficiency is on par with Cas9-mediated HDR.

We also noticed that the distance between the mutation and the AsCas12a target site affects LAHR editing efficiency, which is consistent with previous reports describing the effect of the distance between the mutation and nuclease target site upon Cas9 targeting and ssODN-mediated HDR. When the cut site is more than 10 bp removed from the Cas9 target site, HDR efficiency was shown to drop sharply (45). A solution to prevent this drop in efficiency is to extend the size of the flanking homologous arms on the ssODN (46). This principle may also apply in LAHR, but using simultaneous transduction of the AsCas12a RNP and the LAHR template DNA, we observed that repair efficiencies drop with LAHR templates over 100 bp in size, possibly because these have more trouble passing the nuclear envelope. We determined that with an 80-bp homologous arm, a favorable distance between the mutation and the Cas12a target site is between 0 and 20 bp.

Taken together, we believe LAHR adds an attractive tool to the CRISPR toolbox and provides an essential alternative to traditional Cas9-mediated HDR particularly in circumstances where the Cas9-mediated editing is impaired by the lack of a suitable PAM site or efficient guide RNA candidates.

## DATA AVAILABILITY

NGS data have been deposited in ‘Sequence Read Archive’ with the following link: https://www.ncbi.nlm.nih.gov/sra/PRJNA800830.

## Supplementary Material

gkac118_Supplemental_File
