# Supplementary material for: Ligation-assisted homologous recombination enables precise genome editing by deploying both MMEJ and HDR
Source: Nucleic Acids Res. 2022 Feb 25;50(11):e62. doi: 10.1093/nar/gkac118 (PMC9226534; doi:10.1093/nar/gkac118)
Supplement: gkac118_Supplemental_File [file gkac118_supplemental_file.pdf]

SUPPLEMENTARY FIGURES AND TABLES

Supplementary figure 1

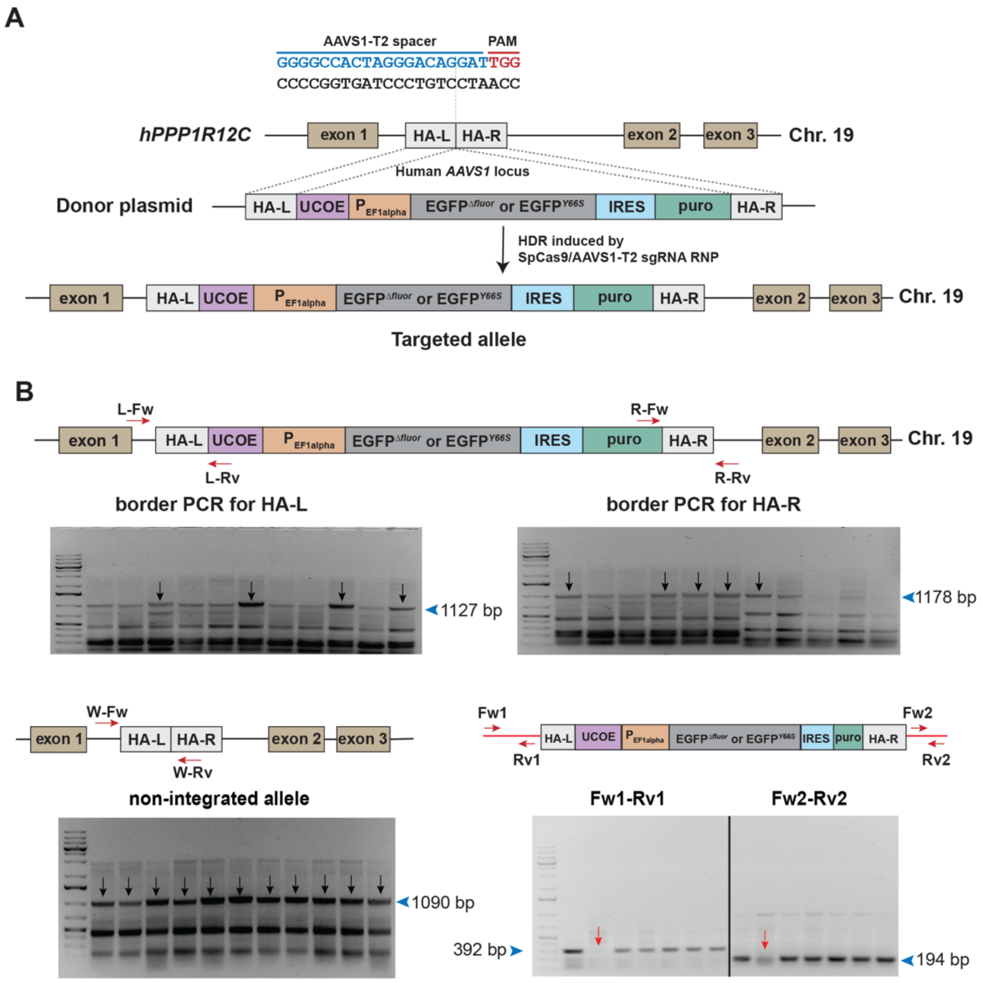

C

Donor plasmid	Survived clones*	Correctly integrated clones**	Heterozygous clones/ Homozygous clones	Single copy clones***
pAAVS1-EGFP$^{\Delta fluor}$	73	45	41/4	1
pAAVS1-EGFP^{Y66S}	64	41	35/6	3

<u>Supplementary figure 1</u> Building the single-copy EGFP$^{\Delta fluor}$ / EGFP^{Y66S} reporter HAP1 cell lines(A) Schematic of the human *AAVS1* locus and two homologous arms (HA-L and HA-R) used to build the donor plasmids. The AAVS1-T2 spacer spanned the border between HA-L and HA-R regions on the genome. The corresponding single guide RNA (sgRNA) targeting the AAVS1-T2 spacer sequence together with SpCas9 protein and donor plasmid were electroporated into HAP1 cells. SpCas9-induced DNA DSB facilitated the HDR using the donor plasmid as repair template. The correctly targeted allele contained a UCOE (Ubiquitous Chromatin Opening Element) sequence, a human EF1alpha promoter, an EGFP$^{\Delta fluor}$ or EGFP^{Y66S} reporter gene, an IRES (Internal Ribosome Entry Site) sequence and a puromycin resistant gene, which were flanked by two homologous arms. **(B)** To verify the correct single

allele targeting, we utilized a PCR based strategy. First, the PCR product across the HA-L border (using the primer pair L-Fw/L-Rv) verified the correct homologous recombination of HA-L, which resulted in a 1127-bp band on the gel image (black arrows represented the positive integrations). The HA-L-integrated clones were selected to proceed with the HA-R border PCR analysis using the primer pair R-Fw/R-Rv, and a 1178-bp band indicated a correct integration of HA-R. Subsequently, the double positive clones from the border PCR analysis were further screened by another round of PCR using the primer set W-Fw and W-Rv, which selected the clones containing a non-integrated allele (a 1090-bp band on the gel image). Finally, two primer sets, Fw1/Rv1 and Fw2/Rv2, targeting two small regions located on the donor plasmid outside the 'HA-L-reporter-HA-R' region were used to exclude the random integration of the donor plasmid. The 392-bp and 194-bp bands indicated the corresponding plasmid regions were randomly integrated in the genome, whereas the double negative clones (red arrows) from this analysis were finally selected as the correct single-copy reporter clones. The gel images shown here were selected to represent the principle. **(C)** Clone screening. * Survived clones from a full 96-well plate of single cells from single-cell sorting, ** Double positive clones from a border PCR screen, *** Single-copy reporter clones from a PCR-based zygosity screen. PCR primers were listed in Supplementary table 1.

Supplementary figure 2

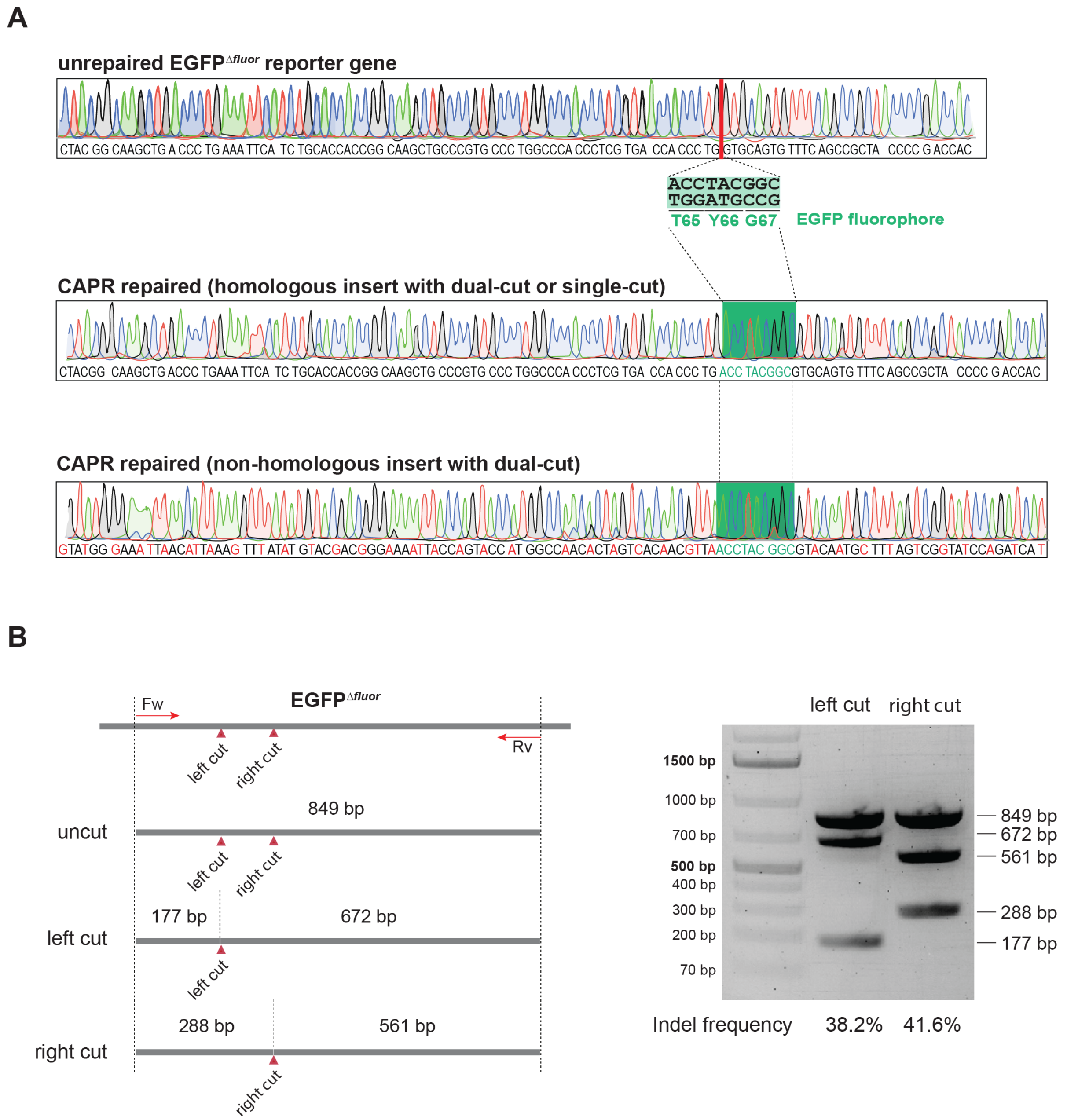

Supplementary figure 2

(A) Verifying CAPR by Sanger sequencing

To confirm the repair of EGFP$^{\Delta fluor}$ reporter cells by CAPR (including 'single-cut' controls), EGFP positive cells were FACS-sorted in both bulk and single cells. Genomic DNA was isolated from both bulk EGFP positive cells and from 20 EGFP-positive clones respectively. The target locus on the genomic DNA then was amplified by PCR, and the PCR products were further analyzed by Sanger sequencing. Chromatograms, from top to bottom, exhibited unrepaired fluorophore-removal locus of the EGFP$^{\Delta fluor}$ reporter gene, the EGFP fluorophore restored by the homologous repair insert with dual or single

AsCas12a cleavage and the repair using a non-homologous repair insert. In the chromatograms showing the repaired EGFP$^{\Delta fluor}$ reporter genes, the recovered EGFP fluorophore coding region was covered by green shadow. Because all the sequencing data did not show mutations, we only present one chromatogram from each group.

(B) Indel efficiencies of AsCas12a-created 'left cut' and 'right cut' on the EGFP$^{\Delta fluor}$ reporter gene
To assess the indel efficiencies of two AsCas12a cleavage sites, 'left cut' and 'right cut', on the EGFP$^{\Delta fluor}$ reporter gene (we used these two sites in CAPR studies. Figure 1). AsCas12a protein and crRNA targeting each site were respectively transduced into the single-copy EGFP$^{\Delta fluor}$ reporter cells. Following genomic DNA isolation, the PCR amplified targeting locus was used to perform T7E1 assay. The schematic displays the predicted cleavage patterns of uncut, left-cut and right-cut PCR products after T7 endonuclease treatment. The agarose gel showed T7 endonuclease-resulting fragments. Band sizes are indicated at the right side of the gel image. The indel efficiencies were quantified using ImageJ analysis of the gel image.

Supplementary figure 3

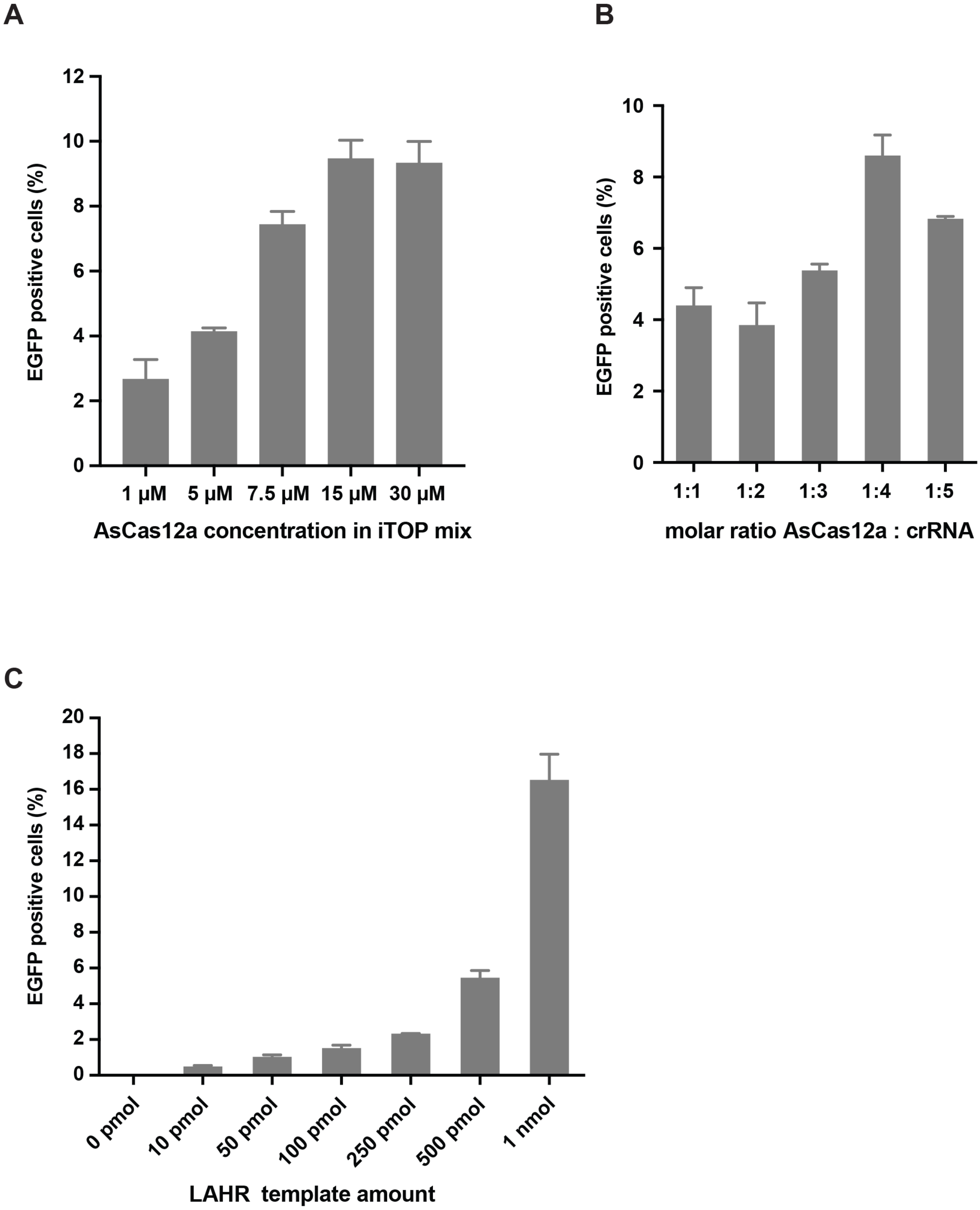

<u>Supplementary figure 3</u> **Optimizing quantities and ratios of LAHR components**

(A) To examine how AsCas12a protein concentration influenced the LAHR efficiency, we tested a AsCas12a protein gradient using the EGFP^{Y66S} mutant reporter cells. At a final concentration of 15 µM

AsCas12a, LAHR efficiency plateaued. Error bars correspond to the standard deviation of the average of n = 3 parallel samples. The experiment was repeated three times and a representative dataset is presented here. **(B)** With different molar ratios between AsCas12 protein and crRNA, LAHR was performed to repair the $EGFP^{Y66S}$ mutation. The experiment demonstrates that 1:4 is the optimized ratio. Error bars corresponded to the standard deviation of the average of n = 3 parallel samples. The experiment was repeated three times and a representative dataset was presented here. **(C)** The repair efficiency showed a positive correlation with the increasing amount of LAHR template. Error bars corresponded to the standard deviation of the average of n = 3 parallel samples. The experiment was repeated three times and a representative dataset was presented here.

Supplementary figure 4

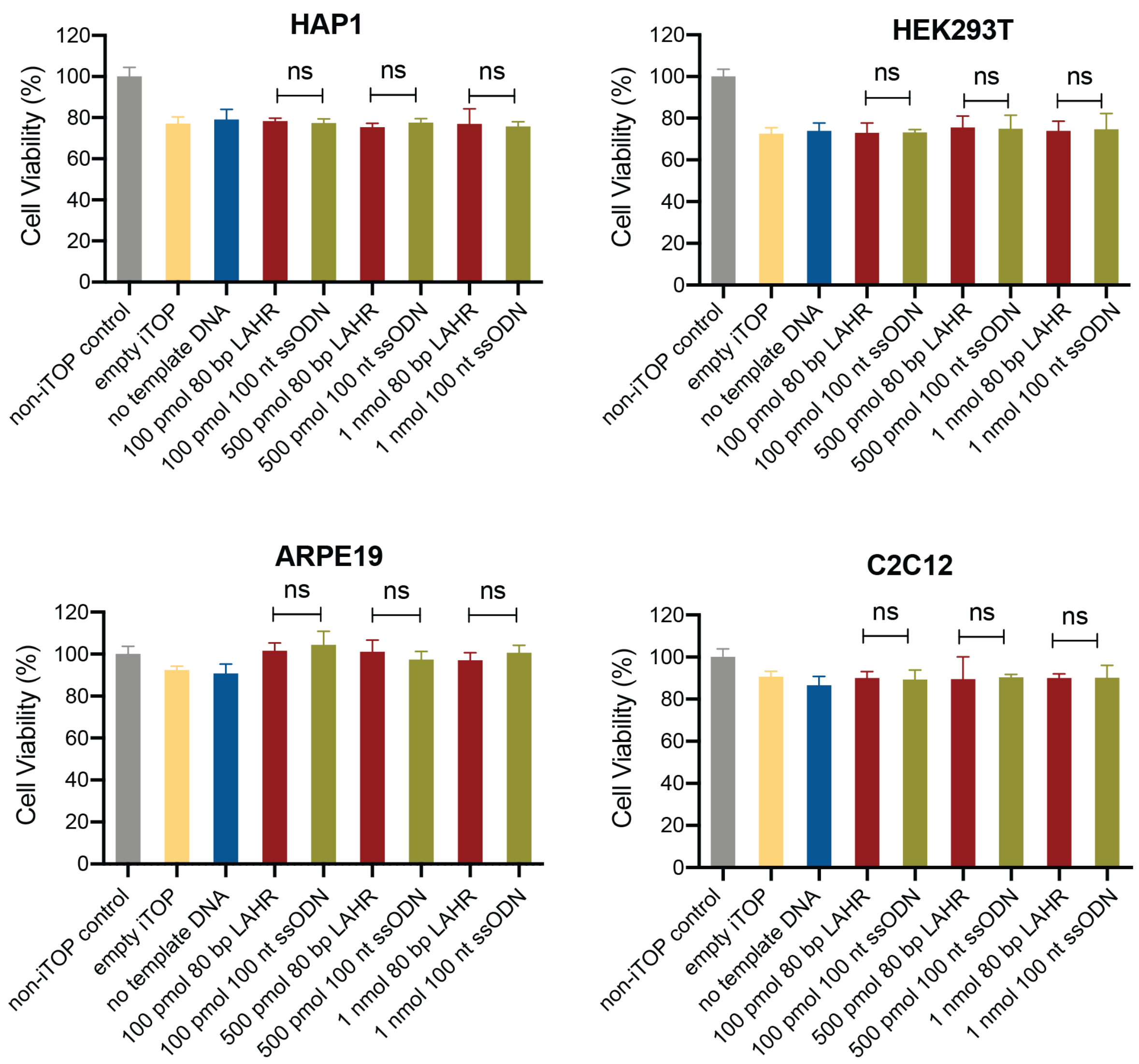

<u>Supplementary figure 4</u> Cell viability assessment following iTOP transduction of LAHR components

The post-iTOP cell viabilities of different cell lines were assessed by MTS assay. The MTS assay was performed 24 hours after iTOP transduction. The bar graphs show the viabilities of different cell lines that performed iTOP deliveries of 80-bp LAHR or 100-nt ssODN template in different quantities, together with AsCas12a protein and crRNA. We observed no significant difference in cell viability between the empty iTOP and the no-template DNA controls and any of the tested template samples. Error bars corresponded to the standard deviation of the average of n = 3 parallel samples. The experiment was repeated three times and a representative dataset was presented here. Statistical test: two-tailed unpaired *t-test*, ns $P > 0.05$.

Supplementary figure 5

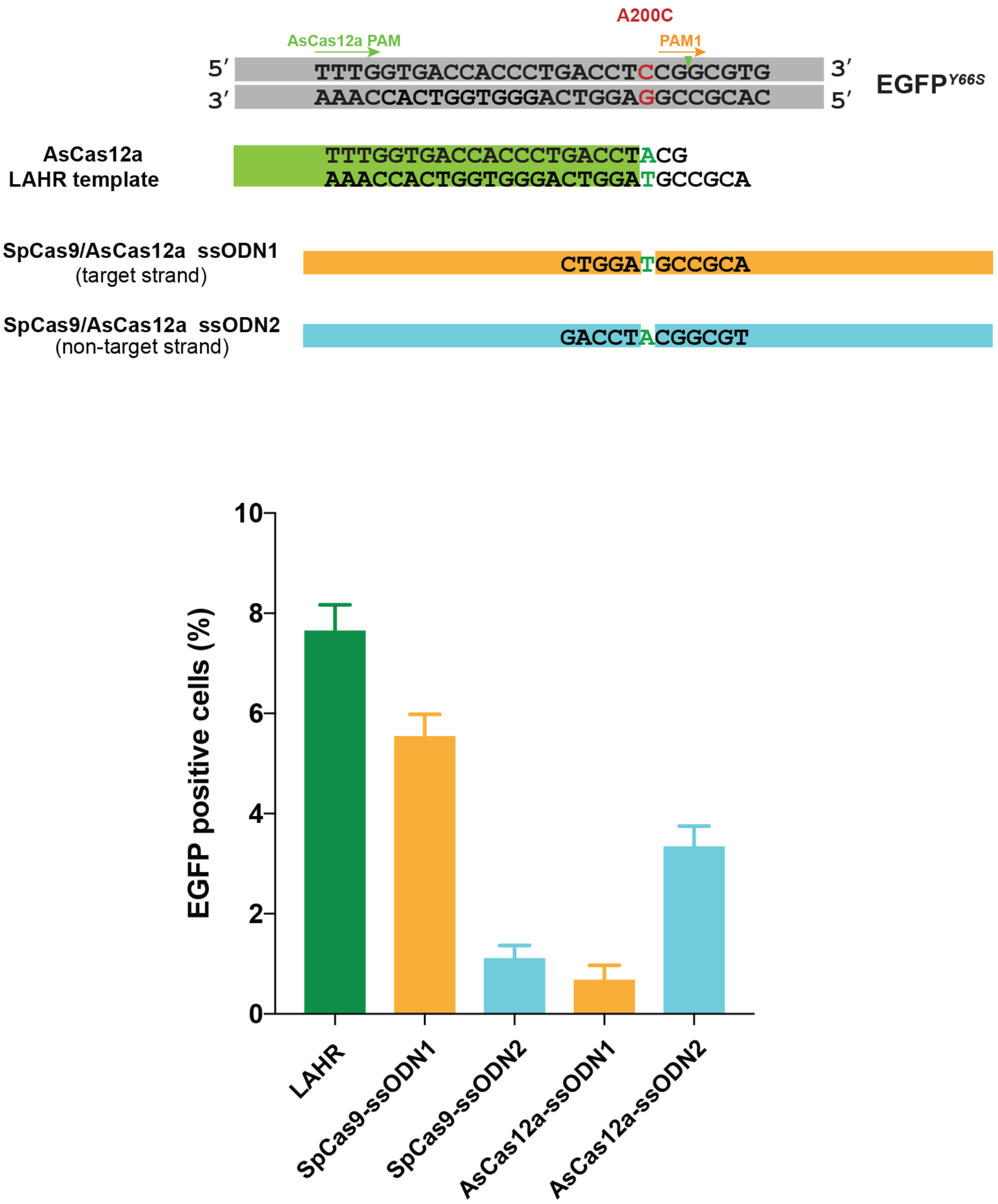

<u>Supplementary figure 5</u> Supplementary comparisons for Figure 3A

Here, we examined HDR efficiency using reverse complementary ssODN templates (ssODN1 and ssODN2) in the EGFP^{Y66S} reporter cell line. Since the AsCas12a PAM (green arrow) and SpCas9 PAM (orange arrow) had the same orientation, for both, the top strand was the 'non-target strand' and the bottom strand was a 'target strand'. The ssODN1 from the 'target strand' favored the Cas9-mediated HDR, while the ssODN2 from the 'non-target strand' favored Cas12a-mediated HDR. This result is consistent with the previously published data (1,2). Error bars corresponded to the standard deviation of the average of n = 3 parallel samples. The experiment was repeated three times and a representative dataset is presented here.

Supplementary figure 6

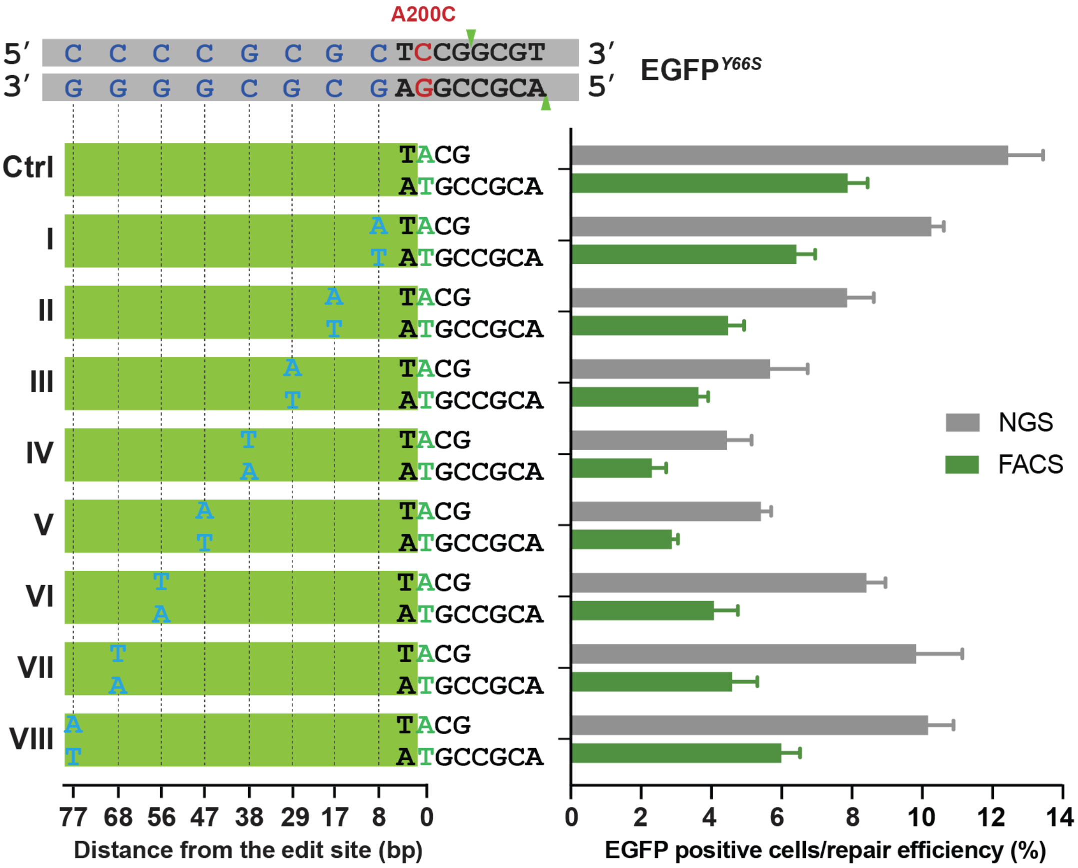

<u>Supplementary figure 6</u> FACS and NGS analyses for LAHR in repairing the 'A200C' mutation

The 'A200C' mutation that turned EGFP off in the EGFP^{Y66S} reporter cells is indicated as a red C/G pair. LAHR templates I to VIII contained base substitutions (blue base pairs) to introduce silent mutations. FACS data (Green bars) showed the percentages of EGFP positive cells after LAHR. The gray bars indicate the repair efficiency of the A200C mutation using the indicated templates. Error bars correspond to the standard deviation of the average of n = 3 parallel samples.

Supplementary figure 7

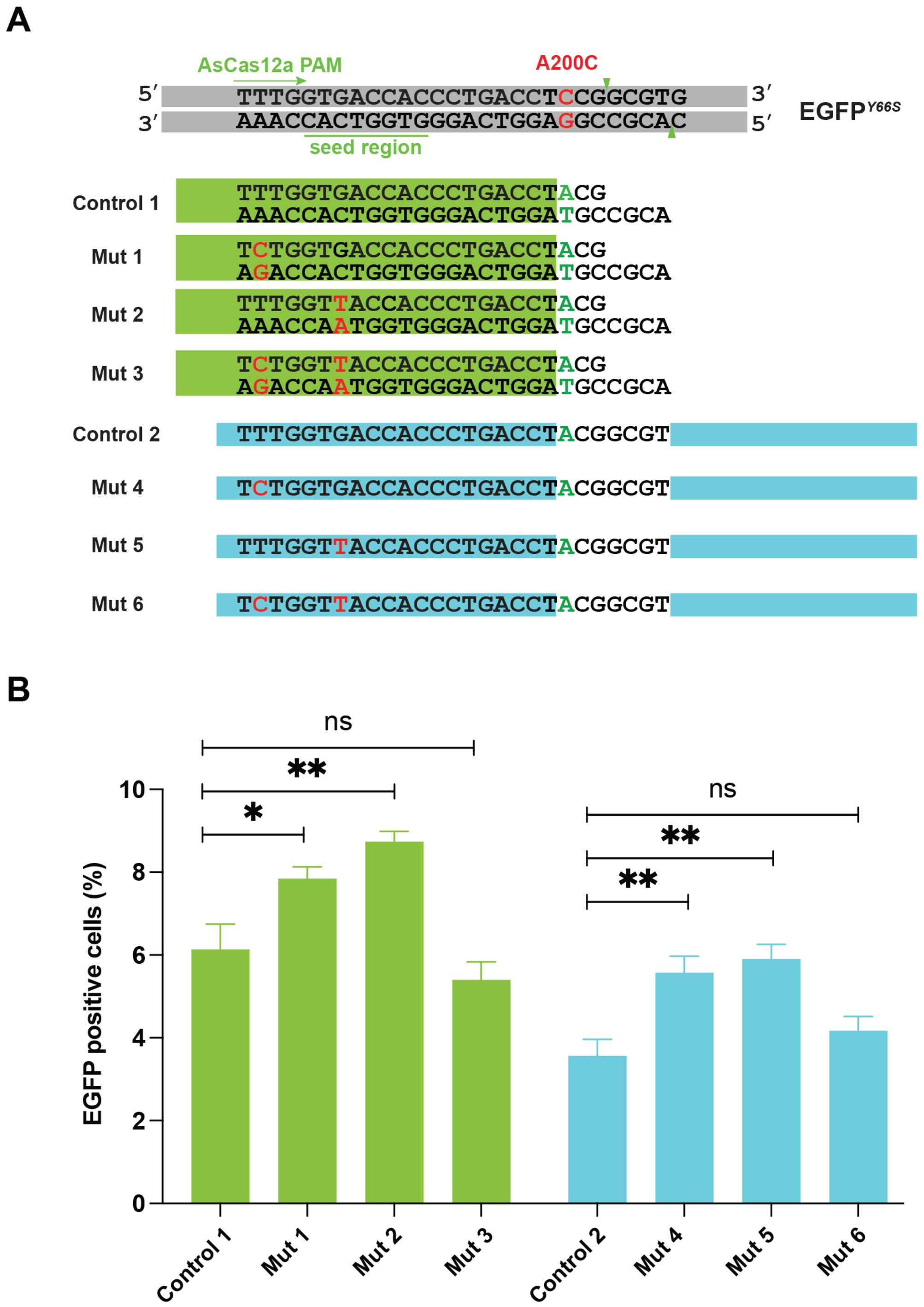

<u>Supplementary figure 7</u> Improving LAHR efficiency by PAM sequence or seed region disruption
(A) In this study, we used the EGFP^{Y66S} reporter cell line described above. The targeting locus is indicated as a stretch of gray-shadowed dsDNA, and the A200C mutation is shown as a red C/G pair. The AsCas12a PAM sequence is indicated by a green arrow on top, and the seed region is green-

underlined. To examine the effects of PAM sequence or seed region disruption on LAHR editing efficiency, we designed different LAHR templates containing silent mutations to destroy PAM sequence (Mut 1), seed region (Mut 2) and both (Mut 3) as indicated. The 'Control 1' template was a LAHR template with unchanged PAM and seed sequences. In addition, for comparison, we also included ssODN templates containing same mutations as those in the LAHR templates, which were 'Mut 4' with mutated PAM, 'Mut 5' with mutated seed and 'Mut 6' with both. The 'Control 2' template was an unmutated ssODN template. **(B)** The corresponding A200C repair efficiencies are indicated by the percentage of EGFP positive cells based on FACS analysis. Error bars correspond to the standard deviation of the average of n = 3 parallel samples.

Supplementary figure 8

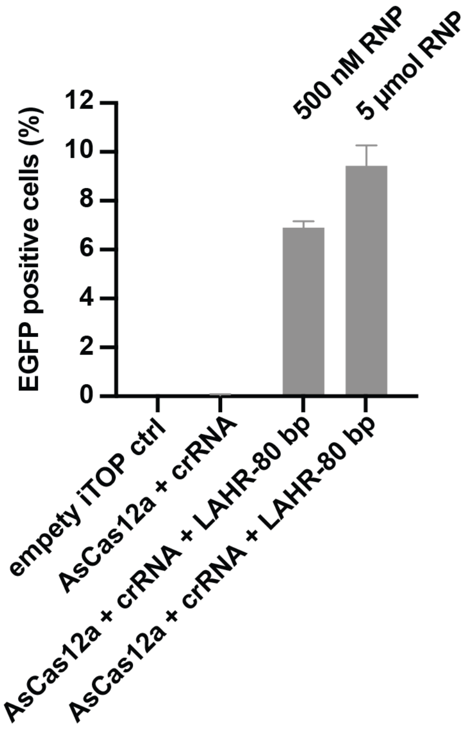

<u>Supplementary figure 8</u> **Delivering Cas12a RNP and LAHR template with electroporation**

To examine the applicability of LAHR with a non-iTOP delivery method, we electroporated AsCas12a RNP and LAHR template into the single-copy EGFP^{Y66S} cells. We tested the LAHR efficiency in two quantity setups, 50 pmol and 500 pmol, and the molar ratio between components is 1:1. The graph depicts the percentage of EGFP-positive cells from the FACS analysis. Error bars correspond to the standard deviation of the average of n = 3 parallel samples. The experiment was repeated three times and a representative dataset is presented here.

Supplementary figure 9

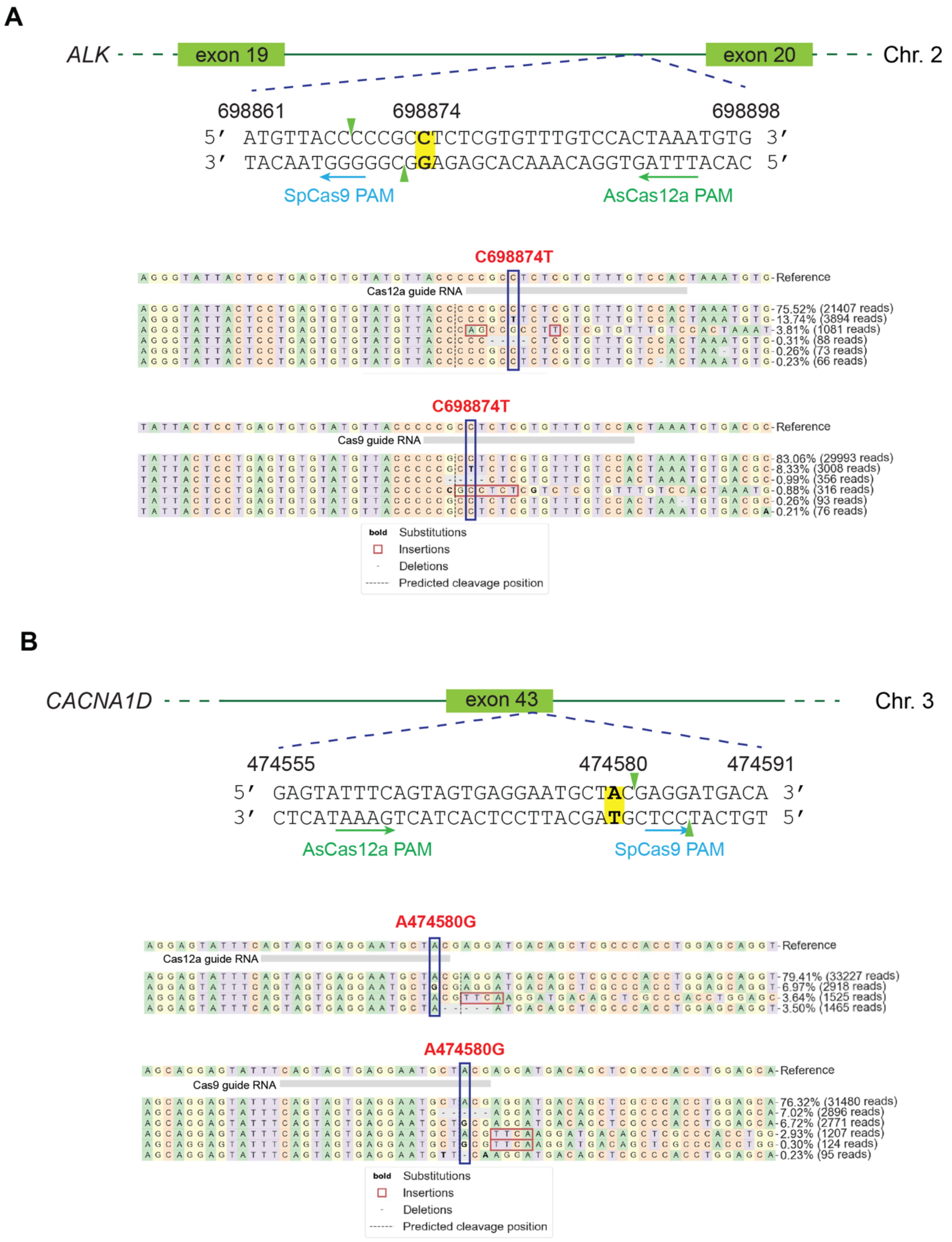

<u>Supplementary figure 9</u> Introducing single nucleotide substitutions in endogenous genes by LAHR and HDR

(A) A single nucleotide substitution, C698874T, was introduced in human *ALK* by LAHR or Cas9-mediated HDR**.** The editing locus, between 698861 and 698898, was in the intron 19 of the ALK gene, and is shown as double-stranded DNA. The AsCas12a PAM is indicated by a green arrow. Green arrow heads indicate the AsCas12a cleavage sites. The yellow-shadowed base pair is the base substitution target. The SpCas9 PAM is indicated by a blue arrow. The alignments below the schematic are the NGS results. Guide RNA targeting sequences are underlined by grey bars. The C698874T substitution is highlighted by a blue box. The percentage of the total reads and the number of reads (in brackets) is shown at the end of each edited sequence. **(B)** A single nucleotide substitution, A474580G, was introduced in human *CACNA1D* by LAHR or Cas9-mediated HDR**.** The editing locus, between 474555 and 474591, was in the exon 43 of the CACNA1D gene, and is shown as double-stranded DNA. The AsCas12a PAM is indicated by a green arrow. Green arrow heads indicate the AsCas12a cleavage sites. The yellow-shadowed base pair is the base substitution target. The SpCas9 PAM is indicated by a blue arrow. The alignments below the scheme are the NGS results. Guide RNA targeting sequences are underlined by grey bars. The A474580G substitution is highlighted by a blue box. The percentage of the total reads and the number of reads (in brackets) is shown at the end of each edited sequence.

Supplementary figure 10

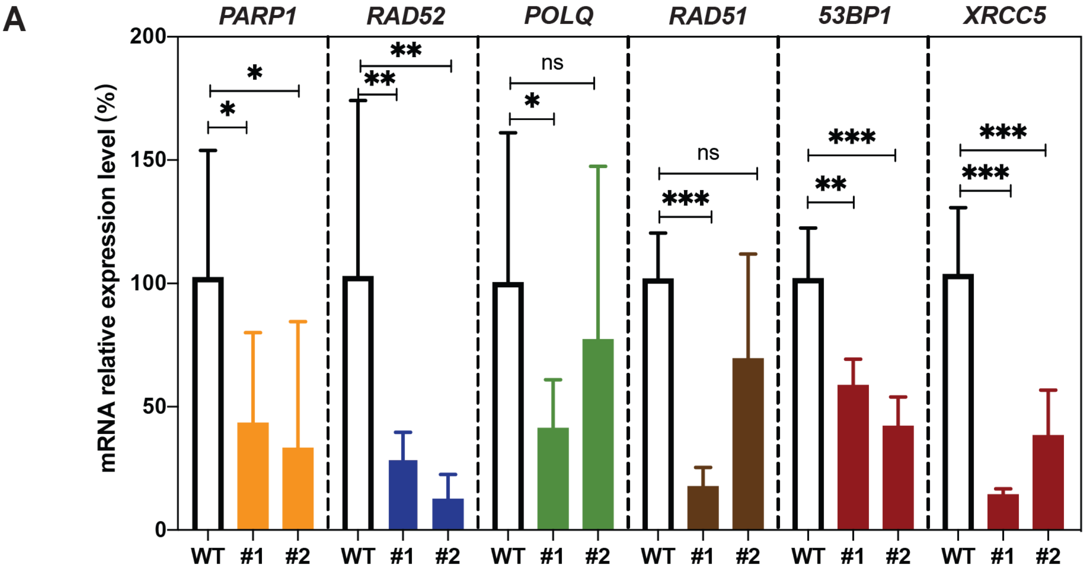

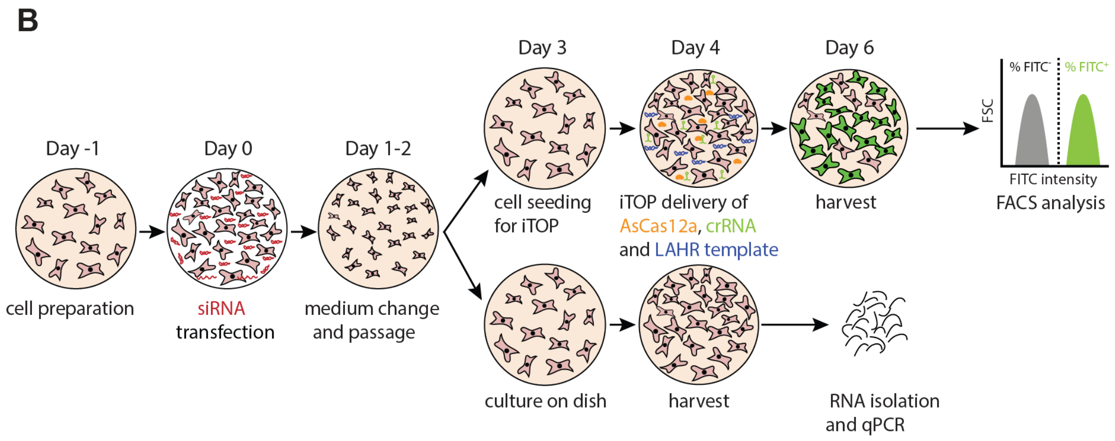

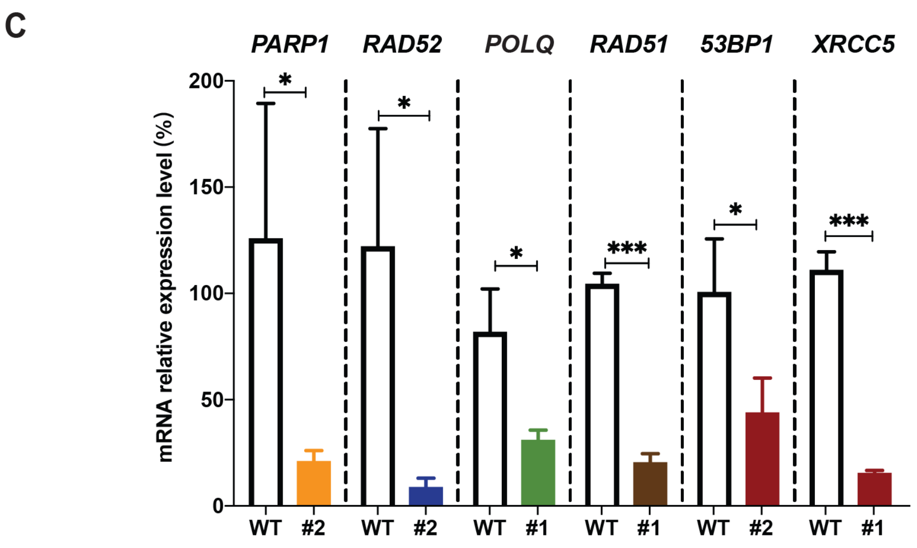

Supplementary figure 10

(A) Selection of the siRNA achieving efficient knockdown

For each target gene, two siRNAs (#1 and #2) were tested. The bar graphs show the expression level of each target gene 48 hours after transfection. 'WT' indicates the expression level of the target gene in an siRNA-free control transfection sample. Error bars correspond to the standard deviation of the average of 3 biological replicate groups, in each of which 3 technical replicates were included. Statistical test: two-tailed unpaired t-test, ns $P > 0.05$, * $P < 0.05$, ** $P < 0.01$, *** $P < 0.001$.

(B) Experimental setup of LAHR under siRNA knockdown conditions

Schematic representation of the experimental setup. In brief, siRNA transfections (using selected siRNAs from (A)) were performed on day 0. Two days after, each siRNA-transfected sample was split into two plates. One group of plates was prepared for the iTOP transduction on day 4, followed by FACS analysis on day 6 to determine the repair efficiencies under different knockdown conditions, while cells on the other group of plates were cultured till day 6 for RNA isolation and the qPCR analysis to examine the knockdown efficacies of the target genes at the time point when LAHR were applied.

(C) Confirmation of the siRNA-knockdown efficacy by qPCR

Analysis of the knockdown efficacies of target genes at the time point of LAHR editing by qPCR. The bar graphs demonstrate the gene expression level. 'WT' indicated the expression level of the target gene in an siRNA-free control transfection sample. Error bars correspond to the standard deviation of the average of 3 technical replicates. Statistical test: two-tailed unpaired t-test, * $P < 0.05$, *** $P < 0.001$.

Supplementary figure 11

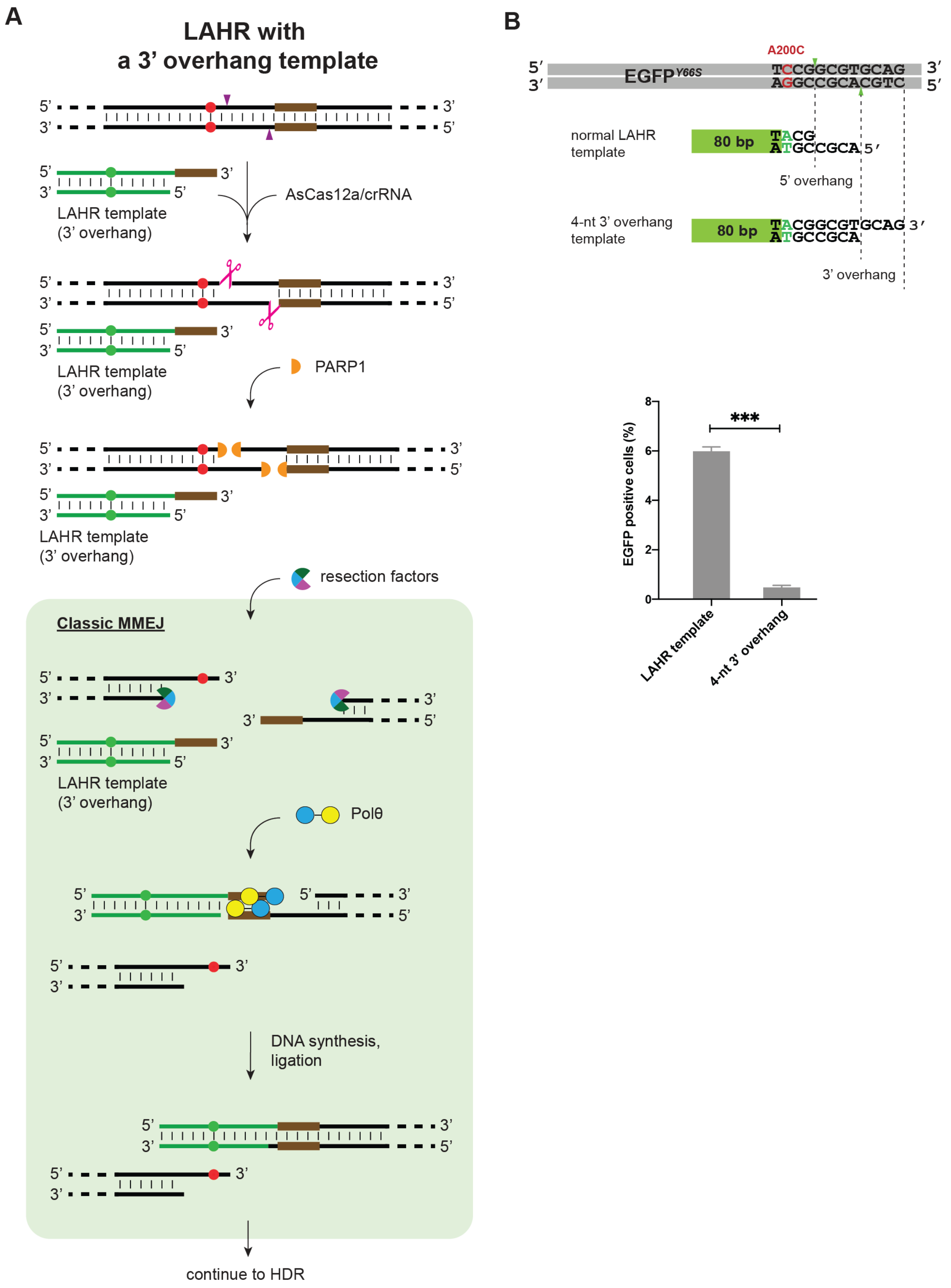

Supplementary figure 11 Repairing the EGFP^{Y66S} mutant by LAHR following a classic-MMEJ pathway

(A) Schematic representation of a LAHR template containing a 3' homologous overhang, which forces the ligation step of LAHR to follow a classic MMEJ pathway. The targeting locus is presented as a stretch of dsDNA containing a mutation (red dots on both strands), the AsCas12a cut site is indicated by purple arrow heads and a homologous region is indicated with brown blocks. The LAHR template (with a 3' overhang) contains a base substitution (green dots on both strands) and a 3' homologous overhang (a brown block). After AsCas12a cleavage, the homologous region on the editing locus was double-stranded, which only matched the 3' homologous overhang on the repair if a compatible 3' homologous overhang is created by resection. In the green box, the classic MMEJ pathway is shown. **(B)** An LAHR template containing a 3' homologous overhang was used to repair the EGFP^{Y66S} mutation. A standard LAHR template (containing a 5' overhang) was used as control. The bar chart shows the comparison of the repair efficiencies as percentage of the EGFP-positive cells determined by flow-cytometry analysis. Error bars correspond to the standard deviation of the average of n = 3 parallel samples. The experiment was repeated three times and a representative dataset is presented here. Statistical test: two-tailed unpaired t-test, *** P < 0.001.

Supplementary figure 12

Figure 2B

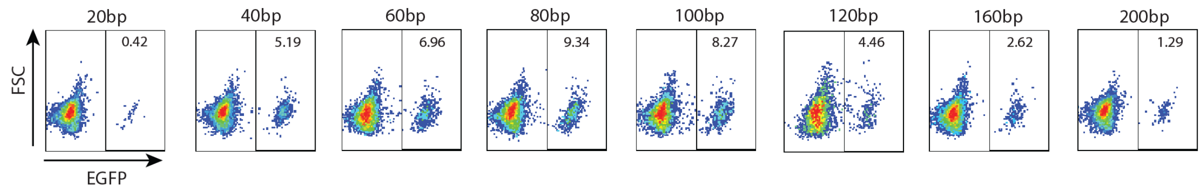

Figure 2C

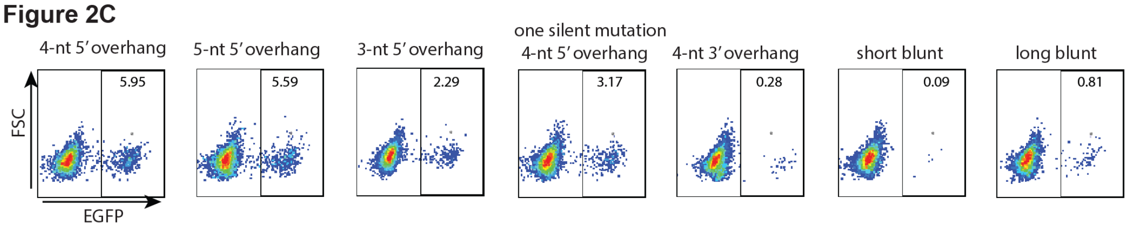

Figure 3A

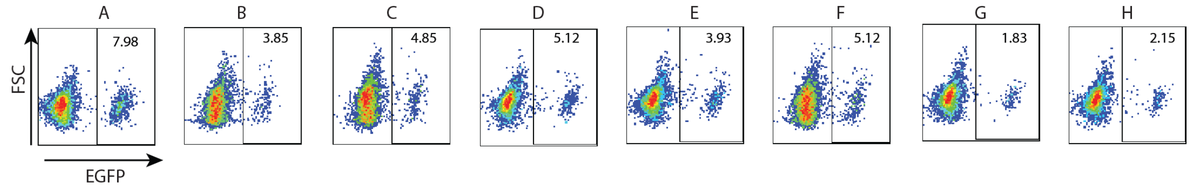

Figure 4B

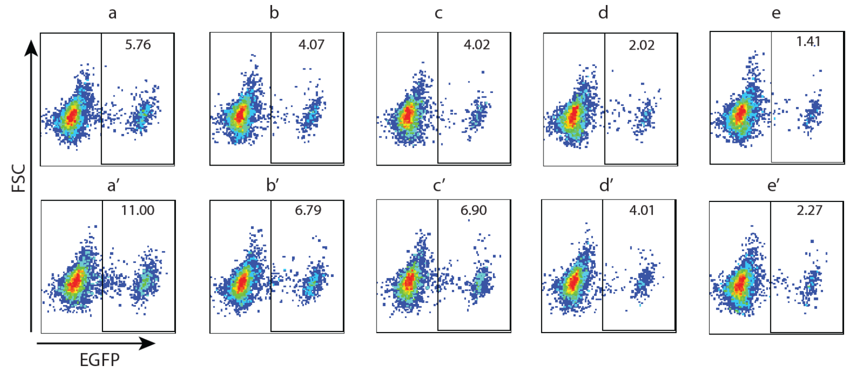

Supplementary figure 6

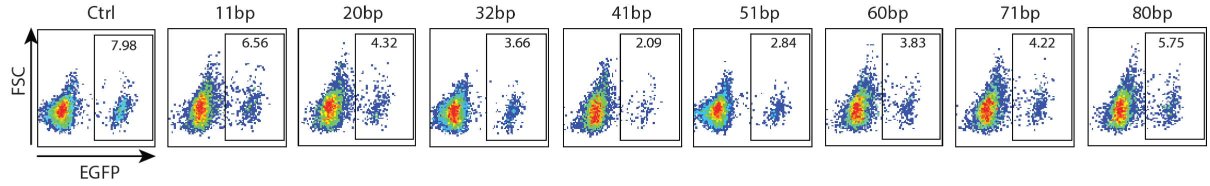

<u>Supplementary figure 12</u> FACS plots of Figure 2, 3, 4 and Supplementary figure 6

Supplementary Table 1 Border-PCR primers

Name	Sequence (5’ to 3’)
L-Fw	TGGACTTTGTCTCCTTCCCTG
L-Rv	GTGGATGAATACTGCCATTTGTG
R-Fw	ATGATCTGTGTGTGTTGGTTT
R-Rv	AGACCTGACCCAAACCCAG
W-Fw	TGGACTTTGTCTCCTTCCCTG
W-Rv	TGGGGCTTTTCTGTCACCAAT
Fw1	GAAGAATCGCAAAACCAGCAAG
Rv1	ATCGAATGGATCTGTCTCTGTC
Fw2	ATTGCCACCACCTGTCAGC
Rv2	GCAGAATCCAGGTGGCAAC

Supplementary Table 2 gBlock fragments of EGFP mutants

Name	Sequence (5’ to 3’)
$EGFP^{\Delta fluor}$	GTGAAGCGGCCGCCACCATGGTGAGCAAGGGCGAGGAGCTGTTCACCGGGGTGGTG<br>CCCATCCTGGTCGAGCTGGACGGCGACGTAAACGGCCACAAGTTCAGCGTGTCCGG<br>CGAGGGCGAGGGCGATGCCACCTACGGCAAGCTGACCCTGAAATTCATCTGCACCAC<br>CGGCAAGCTGCCCGTGCCCTGGCCCACCCTCGTGACCACCCTGGTGCAGTGTTTCAG<br>CCGCTACCCCGACCACATGAAGCAGCACGACTTCTTCAAGTCCGCCATGCCCGAAGG<br>CTACGTCCAGGAGCGCACCATCTTCTTCAAGGACGACGGCAACTACAAGACCCGCGC<br>CGAGGTGAAGTTCGAGGGCGACACCCTGGTGAACCGCATCGAGCTGAAGGGCATCG<br>ACTTCAAGGAGGACGGCAACATCCTGGGGCACAAGCTGGAGTACAACTACAACAGCC<br>ACAACGTCTATATCATGGCCGACAAGCAGAAGAACGGCATCAAGGTGAACTTCAAGAT<br>CCGCCACAACATCGAGGACGGCAGCGTGCAGCTCGCCGACCACTACCAGCAGAACA<br>CCCCCATCGGCGACGGCCCCGTGCTGCTGCCCGACAACCACTACCTGAGCACCCAG<br>TCCGCCCTGAGCAAAGACCCCAACGAGAAGCGCGATCACATGGTCCTGCTGGAGTTC<br>GTGACCGCCGCCGGGATCACTCTCGGCATGGACGAGCTGTACAAGTAA
$EGFP^{Y66S}$	GTGAAGCGGCCGCCACCATGGTGAGCAAGGGCGAGGAGCTGTTCACCGGGGTGGTG<br>CCCATCCTGGTCGAGCTGGACGGCGACGTAAACGGCCACAAGTTCAGCGTGTCCGG<br>CGAGGGCGAGGGCGATGCCACCTACGGCAAGCTGACCCTGAAGTTCATCTGCACCAC<br>CGGCAAGCTGCCCGTGCCCTGGCCCACTTTGGTGACCACCCTGACCTCCGGCGTGC<br>AGTGCTTCAGCCGCTACCCCGACCACATGAAGCAGCACGACTTCTTCAAGTCCGCCAT<br>GCCCGAAGGCTACGTCCAGGAGCGCACCATCTTCTTCAAGGACGACGGCAACTACAA<br>GACCCGCGCCGAGGTGAAGTTCGAGGGCGACACCCTGGTGAACCGCATCGAGCTGA<br>AGGGCATCGACTTCAAGGAGGACGGCAACATCCTGGGGCACAAGCTGGAGTACAACT<br>ACAACAGCCACAACGTCTATATCATGGCCGACAAGCAGAAGAACGGCATCAAGGTGA<br>ACTTCAAGATCCGCCACAACATCGAGGACGGCAGCGTGCAGCTCGCCGACCACTACC<br>AGCAGAACACCCCCATCGGCGACGGCCCCGTGCTGCTGCCCGACAACCACTACCTGA

	GCACCCAGTCCGCCCTGAGCAAAGACCCCAACGAGAAGCGCGATCACATGGTCCTGC TGGAGTTCGTGACCGCCGCCGGGATCACTCTCGGCATGGACGAGCTGTACAAGTAA

Supplementary Table 3 Guide RNAs used in this study

Name	Sequence (5' to 3')
AAVS1 targeting (SpCas9)	GGGGCCACUAGGGACAGGAUGUUUUAGAGCUAGAAAUAGCAAGUUAAA AUAAGGCUAGUCCGUUAUCAACUUGAAAAAGUGGCACCGAGUCGGUGC
CAPR-left (AsCas12a)	UAAUUUCUACUCUUGUAGAUAGGGUCAGCUUGCCGUAGGUGGC
CAPR-right (AsCas12a)	UAAUUUCUACUCUUGUAGAUAGCCGCUACCCCGACCACAUGAA
$EGFP^{Y66S}$ (AsCas12a)	UAAUUUCUACUCUUGUAGAUGUGACCACCCUGACCUCCGGCGU
B2M (AsCas12a)	UAAUUUCUACUCUUGUAGAUAUCCAUCCGACAUUGAAGUUGAC
ALK (AsCas12a)	UAAUUUCUACUCUUGUAGAUGUGGACAAACACGAGAGGCGGGG
CACNA1D (AsCas12a)	UAAUUUCUACUCUUGUAGAUAGUAGUGAGGAAUGCUACGAGGA
PAM1 (SpCas9)	UUGGTGACCACCCUGACCUCGUUUUAGAGCUAGAAAUAGCAAGUUAAA AUAAGGCUAGUCCGUUAUCAACUUGAAAAAGUGGCACCGAGUCGGUGC
PAM2 (SpCas9)	GCUGAAGCACUGCACGCCGGGUUUUAGAGCUAGAAAUAGCAAGUUAAA AUAAGGCUAGUCCGUUAUCAACUUGAAAAAGUGGCACCGAGUCGGUGC
PAM3 (SpCas9)	GCGGCUGAAGCACUGCACGCGUUUUAGAGCUAGAAAUAGCAAGUUAAA AUAAGGCUAGUCCGUUAUCAACUUGAAAAAGUGGCACCGAGUCGGUGC
B2M-PAM1 (SpCas9)	GAAGUUGACUUACUGAAGAAGUUUUAGAGCUAGAAAUAGCAAGUUAAA AUAAGGCUAGUCCGUUAUCAACUUGAAAAAGUGGCACCGAGUCGGUGC
B2M-PAM2 (SpCas9)	CAGTAAGTCAACTTCAATGTGUUUUAGAGCUAGAAAUAGCAAGUUAAAA UAAGGCUAGUCCGUUAUCAACUUGAAAAAGUGGCACCGAGUCGGUGC
B2M-PAM3 (SpCas9)	AAGTCAACTTCAATGTCGGAGUUUUAGAGCUAGAAAUAGCAAGUUAAAA UAAGGCUAGUCCGUUAUCAACUUGAAAAAGUGGCACCGAGUCGGUGC
ALK (SpCas9)	UGGACAAACACGAGAGGCGGGUUUUAGAGCUAGAAAUAGCAAGUUAAA AUAAGGCUAGUCCGUUAUCAACUUGAAAAAGUGGCACCGAGUCGGUGC
CACNA1D (SpCas9)	CAGUAGUAAGGAAUGCUACGGUUUUAGAGCUAGAAAUAGCAAGUUAAA AUAAGGCUAGUCCGUUAUCAACUUGAAAAAGUGGCACCGAGUCGGUGC

Supplementary Table 4 Repair inserts used in Figure 1

EGFP fluorophore coding sequence

Name	Sequence (5’ to 3’)
sticky-ended	GCCACCTACGGCAAGCTGACCCTGAAATTCATCTGCACCACCGGCAAGCTGCCCGTGCCCTGGCCCACCCTCGTGACCACCCTGACCTACGGCGTGCAGTGTTTCAGCCGCTACCCCGACCAC
	TCATGTGGTCGGGGTAGCGGCTGAAACACTGCACGCCGTAGGTCAGGGTGGTCACGAGGGTGGGCCAGGGCACGGGCAGCTTGCCGGTGGTGCAGATGAATTTCAGGGTCAGCTTGCCGTAGG
blunt-ended	GCCACCTACGGCAAGCTGACCCTGAAATTCATCTGCACCACCGGCAAGCTGCCCGTGCCCTGGCCCACCCTCGTGACCACCCTGACCTACGGCGTGCAGTGTTTCAGCCGCTACCCCGACCACATGA
	TCATGTGGTCGGGGTAGCGGCTGAAACACTGCACGCCGTAGGTCAGGGTGGTCACGAGGGTGGGCCAGGGCACGGGCAGCTTGCCGGTGGTGCAGATGAATTTCAGGGTCAGCTTGCCGTAGGTGGC
sticky-ended (non-homologous)	GCCACGTATGGGAAATTAACATTAAAGTTTATATGTACGACGGGAAAATTACCAGTACCATGGCCAACACTAGTCACAACGTTAACCTACGGCGTACAATACTTTAGTCGGTATCCAGATCAT
	TCATATGATCTGGATACCGACTAAAGTATTGTACGCCGTAGGTTAACGTTGTGACTAGTGTTGGCCATGGTACTGGTAATTTTCCCGTCGTACATATAAACTTTAATGTTAATTTCCCATACG

Supplementary Table 5 Repair templates used in Figure 2

Mutation site

Name	Sequence (5’ to 3’)
20 bp	GGTGACCACCCTGACCTACG
	ACGCCGTAGGTCAGGGTGGTCACC
40 bp	CCCGTGCCCTGGCCCACTTTGGTGACCACCCTGACCTACG
	ACGCCGTAGGTCAGGGTGGTCACCAAAGTGGGCCAGGGCACGGG
60 bp	TCTGCACCACCGGCAAGCTGCCCGTGCCCTGGCCCACTTTGGTGACCACCCTGACCTACG
	CGTAGGTCAGGGTGGTCACCAAAGTGGGCCAGGGCACGGGCAGCTTGCCGGTGGTGCAGA
80 bp	CAAGCTGACCCTGAAGTTCATCTGCACCACCGGCAAGCTGCCCGTGCCCTGGCCCACTTTGGTGACCACCCTGACCTACG
	ACGCCGTAGGTCAGGGTGGTCACCAAAGTGGGCCAGGGCACGGGCAGCTTGCCGGTGGTGCAGATGAACTTCAGGGTCAGCTTG

100 bp	GAGGGCGATGCCACCTACGGCAAGCTGACCCTGAAGTTCATCTGCACCACCGGCA<br>AGCTGCCCGTGCCCTGGCCCACTTTGGTGACCACCCTGACCTACG
	ACGCCGTAGGTCAGGGTGGTCACCAAAGTGGGCCAGGGCACGGGCAGCTTGCCG<br>GTGGTGCAGATGAACTTCAGGGTCAGCTTGCCGTAGGTGGCATCGCCCTC
120 bp	TCAGCGTGTCCGGCGAGGGCGAGGGCGATGCCACCTACGGCAAGCTGACCCTGA<br>AGTTCATCTGCACCACCGGCAAGCTGCCCGTGCCCTGGCCCACTTTGGTGACCAC<br>CCTGACCTACG
	ACGCCGTAGGTCAGGGTGGTCACCAAAGTGGGCCAGGGCACGGGCAGCTTGCCG<br>GTGGTGCAGATGAACTTCAGGGTCAGCTTGCCGTAGGTGGCATCGCCCTCGCCCT<br>CGCCGGACACGCTGA
160 bp	ATCCTGGTCGAGCTGGACGGCGACGTAAACGGCCACAAGTTCAGCGTGTCCGGCG<br>AGGGCGAGGGCGATGCCACCTACGGCAAGCTGACCCTGAAGTTCATCTGCACCAC<br>CGGCAAGCTGCCCGTGCCCTGGCCCACTTTGGTGACCACCCTGACCTACG
	ACGCCGTAGGTCAGGGTGGTCACCAAAGTGGGCCAGGGCACGGGCAGCTTGCCG<br>GTGGTGCAGATGAACTTCAGGGTCAGCTTGCCGTAGGTGGCATCGCCCTCGCCCT<br>CGCCGGACACGCTGAACTTGTGGCCGTTTACGTCGCCGTCCAGCTCGACCAGGAT
200 bp	GGTGAGCAAGGGCGAGGAGCTGTTCACCGGGGTGGTGCCCATCCTGGTCGAGCT<br>GGACGGCGACGTAAACGGCCACAAGTTCAGCGTGTCCGGCGAGGGCGAGGGCGA<br>TGCCACCTACGGCAAGCTGACCCTGAAGTTCATCTGCACCACCGGCAAGCTGCCC<br>GTGCCCTGGCCCACTTTGGTGACCACCCTGACCTACG
	ACGCCGTAGGTCAGGGTGGTCACCAAAGTGGGCCAGGGCACGGGCAGCTTGCCG<br>GTGGTGCAGATGAACTTCAGGGTCAGCTTGCCGTAGGTGGCATCGCCCTCGCCCT<br>CGCCGGACACGCTGAACTTGTGGCCGTTTACGTCGCCGTCCAGCTCGACCAGGAT<br>GGGCACCACCCCGGTGAACAGCTCCTCGCCCTTGCTCACC
4-nt 5’ overhang	CAAGCTGACCCTGAAGTTCATCTGCACCACCGGCAAGCTGCCCGTGCCCTGGCCC<br>ACTTTGGTGACCACCCTGACCTACG
	ACGCCGTAGGTCAGGGTGGTCACCAAAGTGGGCCAGGGCACGGGCAGCTTGCCG<br>GTGGTGCAGATGAACTTCAGGGTCAGCTTG
5-nt 5’ overhang	CAAGCTGACCCTGAAGTTCATCTGCACCACCGGCAAGCTGCCCGTGCCCTGGCCC<br>ACTTTGGTGACCACCCTGACCTAC
	ACGCCGTAGGTCAGGGTGGTCACCAAAGTGGGCCAGGGCACGGGCAGCTTGCCG<br>GTGGTGCAGATGAACTTCAGGGTCAGCTTG
3-nt 5’ overhang	CAAGCTGACCCTGAAGTTCATCTGCACCACCGGCAAGCTGCCCGTGCCCTGGCCC<br>ACTTTGGTGACCACCCTGACCTACG
	CGCCGTAGGTCAGGGTGGTCACCAAAGTGGGCCAGGGCACGGGCAGCTTGCCGG<br>TGGTGCAGATGAACTTCAGGGTCAGCTTG
4-nt 5’ overhang (one silent mutation)	CAAGCTGACCCTGAAGTTCATCTGCACCACCGGCAAGCTGCCCGTGCCCTGGCCC<br>ACTTTGGTGACCACCCTGACCTACG
	ACTCCGTAGGTCAGGGTGGTCACCAAAGTGGGCCAGGGCACGGGCAGCTTGCCG<br>GTGGTGCAGATGAACTTCAGGGTCAGCTTG

4-nt 3’ overhang	CAAGCTGACCCTGAAGTTCATCTGCACCACCGGCAAGCTGCCCGTGCCCTGGCCCACTTTGGTGACCACCCTGACCTACGGCGT
	CGTAGGTCAGGGTGGTCACCAAAGTGGCCAGGGCACGGGCAGCTTGCCGGTGGTGCAGATGAACTTCAGGGTCAGCTTG
Short blunt	CAAGCTGACCCTGAAGTTCATCTGCACCACCGGCAAGCTGCCCGTGCCCTGGCCCACTTTGGTGACCACCCTGACCTACG
	CGTAGGTCAGGGTGGTCACCAAAGTGGCCAGGGCACGGGCAGCTTGCCGGTGGTGCAGATGAACTTCAGGGTCAGCTTG
Long blunt	CAAGCTGACCCTGAAGTTCATCTGCACCACCGGCAAGCTGCCCGTGCCCTGGCCCACTTTGGTGACCACCCTGACCTACGGCGT
	ACGCCGTAGGTCAGGGTGGTCACCAAAGTGGCCAGGGCACGGGCAGCTTGCCGGTGGTGCAGATGAACTTCAGGGTCAGCTTG
4-nt 3’ overhang (Supplementary figure 11)	CAAGCTGACCCTGAAGTTCATCTGCACCACCGGCAAGCTGCCCGTGCCCTGGCCCACTTTGGTGACCACCCTGACCTACGGCGTGCAG
	ACGCCGTAGGTCAGGGTGGTCACCAAAGTGGCCAGGGCACGGGCAGCTTGCCGGTGGTGCAGATGAACTTCAGGGTCAGCTTG

Supplementary Table 6 Repair templates used in Figure 3

Mutation site

Name	Sequence (5’ to 3’)
a	CAAGCTGACCCTGAAGTTCATCTGCACCACCGGCAAGCTGCCCGTGCCCTGGCCCACTTTGGTGACCACCCTGACCTACG
	ACGCCGTAGGTCAGGGTGGTCACCAAAGTGGCCAGGGCACGGGCAGCTTGCCGGTGGTGCAGATGAACTTCAGGGTCAGCTTG
b	CGGCAAGCTGACCCTGAAGTTCATCTGCACCACCGGCAAGCTGCCCGTGCCCTGGCCCACTTTGGTGACCACCCTGACCTACGGCGTGCAGTGCTTCAGCCGCTACCCCGACCACATGAAGCAGCACGACTTCTTCAAGTCCGCCATGCCCGAAGGCTAC
	GTAGCCTTCGGGCATGGCGGACTTGAAGAAGTCGTGCTGCTTCATGTGGTCGGGGTAGCGGCTGAAGCACTGCACGCCGTAGGTCAGGGTGGTCACCAAAGTGGCCAGGGCACGGGCAGCTTGCCGGTGGTGCAGATGAACTTCAGGGTCAGCTTGCCG
c	CGGCAAGCTGACCCTGAAGTTCATCTGCACCACCGGCAAGCTGCCCGTGCCCTGGCCCACTTTGGTGACCACCCTGACCTACGGCGTGCAGTGCTTCAGCCGCTACCCCGACCACATGAAGCAGCACGACTTCTTCAAGTCCGCCATGCCCGAAGGCTAC
d	CGGCAAGCTGCCCGTGCCCTGGCCCACTTTGGTGACCACCCTGACCTACG
	ACGCCGTAGGTCAGGGTGGTCACCAAAGTGGCCAGGGCACGGGCAGCTTGCCG
e/g/h	CACCGGCAAGCTGCCCGTGCCCTGGCCCACTTTGGTGACCACCCTGACCTACGGCGTGCAGTGCTTCAGCCGCTACCCCGACCACATGAAGCAGCACGACT
f	AGTCGTGCTGCTTCATGTGGTCGGGGTAGCGGCTGAAGCACTGCACGCCGTAGGTCAGGGTGGTCACCAAAGTGGCCAGGGCACGGGCAGCTTGCCGGTG

I	CAAGCTGACCCTGAAGTTCATCTGCACCACCGGCAAGCTGCCCGTGCCCTGGCCCACTTT<br>GGTGACCACCCTGACCTACG
	ACGCCGTAGGTCAGGGTGGTCACCAAAGTGGGCCAGGGCACGGGCAGCTTGCCGGTGG<br>TGCAGATGAACTTCAGGGTCAGCTTG
II	CAAGCTGACCCTGAAGTTCATCTGCACCACCGGCAAGCTGCCCGTGCCCTGGCCCACTTT<br>GGTGACCACACTGACCTACG
	ACGCCGTAGGTCAGTGTGGTCACCAAAGTGGGCCAGGGCACGGGCAGCTTGCCGGTGG<br>TGCAGATGAACTTCAGGGTCAGCTTG
III	CAAGCTGACCCTGAAGTTCATCTGCACCACCGGCAAGCTGCCCGTGCCCTGGCCCACTT<br>AGGTGACCACCCTGACCTACG
	ACGCCGTAGGTCAGGGTGGTCACCTAAGTGGGCCAGGGCACGGGCAGCTTGCCGGTGG<br>TGCAGATGAACTTCAGGGTCAGCTTG
IV	CAAGCTGACCCTGAAGTTCATCTGCACCACCGGCAAGCTGCCCGTGCCCTTGCCCACTTT<br>GGTGACCACCCTGACCTACG
	ACGCCGTAGGTCAGGGTGGTCACCAAAGTGGGCAAGGGCACGGGCAGCTTGCCGGTGG<br>TGCAGATGAACTTCAGGGTCAGCTTG
V	CAAGCTGACCCTGAAGTTCATCTGCACCACAGGCAAGCTGCCCGTGCCCTGGCCCACTTT<br>GGTGACCACCCTGACCTACG
	ACGCCGTAGGTCAGGGTGGTCACCAAAGTGGGCCAGGGCACGGGCAGCTTGCCTGTGG<br>TGCAGATGAACTTCAGGGTCAGCTTG
VI	CAAGCTGACCCTGAAGTTCATATGCACCACCGGCAAGCTGCCCGTGCCCTGGCCCACTTT<br>GGTGACCACCCTGACCTACG
	ACGCCGTAGGTCAGGGTGGTCACCAAAGTGGGCCAGGGCACGGGCAGCTTGCCGGTGG<br>TGCATATGAACTTCAGGGTCAGCTTG
VII	CAAGCTGACTCTGAAGTTCATCTGCACCACCGGCAAGCTGCCCGTGCCCTGGCCCACTTT<br>GGTGACCACCCTGACCTACG
	ACGCCGTAGGTCAGGGTGGTCACCAAAGTGGGCCAGGGCACGGGCAGCTTGCCGGTGG<br>TGCAGATGAACTTCAGAGTCAGCTTG
VIII	AAAGCTGACCCTGAAGTTCATCTGCACCACCGGCAAGCTGCCCGTGCCCTGGCCCACTTT<br>GGTGACCACCCTGACCTACG
	ACGCCGTAGGTCAGGGTGGTCACCAAAGTGGGCCAGGGCACGGGCAGCTTGCCGGTGG<br>TGCAGATGAACTTCAGGGTCAGCTTT

Supplementary Table 7 Repair templates used in Figure 4

Mutation site

Name	Sequence (5' to 3')
LAHR template (*B2M*)	TCACGTCATCCAGCAGAGAATGGAAAGTCAAATTTCCTGAATTGCTATGTG<br>TCTGGGTTTCATCCATCCGACATTGAAGT
	GTCAACTTCAATGTCGGATGGATGAAACCCAGACACATAGCAATTCAGG<br>AAATTTGACTTTCCATTCTCTGCTGGATGACGTGA

ssODN1 (*B2M*)	AGTCAAATTTCCTGAATTGCTATGTGTCTGGGTTTCATCCATCCGACATTGAAGTTGACTTACTGAAGAATGGAGAGAGAATTGAAAAAGTGGAGCATTC
ssODN2 (*B2M*)	GAATGCTCCACTTTTTCAATTCTCTCTCCATTCTTCAGTAAGTCAACTTCAATGTCGGATGGATGAAACCCAGACACATAGCAATTCAGGAAATTTGACT
LAHR template (*ALK*)	CCCCGCTTCTCGTGTTTGTCCACTAAATGTGACGCCCAGGCTCAGGACCCCCAGCTGCCTCATTATTGTGGCCTGTTTGACTCT
	AGAGTCAAACAGGCCACAATAATGAGGCAGCTGGGGGTCCTGAGCCTGGGCGTCACATTTAGTGGACAAACACGAGAAGC
ssODN (*ALK*)	AGCAAAGCCATGTTGAGGGTATTACTCCTGAGTGTGTATGTTACCCCCGCTTCTCGTGTTTGTCCACTAAATGTGACGCCCAGGCTCAGGACCCCCAGCT
LAHR template (*CACNA1D*)	AGACCCAGAGATACATGGCTATTTCAGGGACCCCCACTGCTTGGGGGAGCAGGAGTATTTCAGTAGTGAGGAATGCTGCG
	TCCTCGCAGCATTCCTCACTACTGAAATACTCCTGCTCCCCCAAGCAGTGGGGGTCCCTGAAATAGCCATGTATCTCTGGGTCT
ssODN (*CACNA1D*)	AGGAGCCAGAGCAGCTCACCTGCTCCAGGTGGGCGAGCTGTCATCCTCGCAGCATTCCTCACTACTGAAATACTCCTGCTCCCCCAAGCAGTGGGGGTCC

Supplementary Table 8 siRNA target sequences and qPCR primers

**Name**	**Sequence (5' to 3')**
T-53BP1#1	GCCAGGUUCUAGAGGAUGA
T-53BP1#2	GCACAAGAACTTATGGAAAGU
T-XRCC5#1	GAAGUUCUGUCACAGCUGA
T-XRCC5#2	GUCUUCAAGGGUGUCUGUC
T-POLQ#1	CCUUAAGACUGUAGGUACU
T-POLQ#2	CAAACAAACCCUUAUCGUAAA
T-PARP1#1	AAGCCUCCGCUCCUGAACAAU
T-PARP1#2	AAGAUAGAGCGUGAAGGCGAA
T-RAD51#1	GGGAAUUAGUGAAGCCAAA
T-RAD51#2	GAAUUGAGACUGGAUCUAU
T-RAD52#1	GGGAAUUAGUGAAGCCAAA
T-RAD52#2	AGUCCAAGGCUUUAUCUUU
53BP1#1-Fw	AGATGGACCCTACTGGAAGTC
53BP1#1-Rv	TGTTCATTGAACCCACTATTACCGTC
53BP1#2-Fw	AGCAAGGACATCCCTGTGACA
53BP1#2-Rv	ACCTCTGACCAGAGAGCTGCA
XRCC5#1-Fw	TCAGAAGAGCAGCGCTTTAACAA
XRCC5#1-Rv	TCGTCCACATCACCACCTTC

XRCC5#2-Fw	AAGACTTGCGGCAATACATG
XRCC5#2-Rv	CAGCATATTCCAAATATGCTGC
POLQ#1-Fw	CAACAGATGGCAACTGAAAATG
POLQ#1-Rv	CTTGTTTCAGGAACTGGAAGAC
POLQ#2-Fw	CATGACAGAGACAGTGAAGAATTG
POLQ#2-Rv	ACTTTGGAGCATACCCTCTC
PARP1#1-Fw	ATACTCCATCCTCAGTGAGGTC
PARP1#1-Rv	ATGGGATCCTTGCTGCTATC
PARP1#2-Fw	GAGATTCTGAAGAAGCCGAGA
PARP1#2-Rv	CAACTTCTCCCAACAGGATTAA
RAD51#1-Fw	AGTGTGGCATAAATGCCAA
RAD51#1-Rv	GTGAAACCCATTGGAACT
RAD51#2-Fw	AGTTCCAATGGGTTTCAC
RAD51#2-Rv	TGGCAGGTGACAGCTA
RAD52#1-Fw	ACAGCACTCCTGTAACTGTCT
RAD52#1-Rv	TGTTGTGCGTTGGTCAGCG
RAD52#2-Fw	GTTGACCTCAACAATGGC
RAD52#2-Rv	CTGGCGTGGAAGCTTATTTA
GAPDH-Fw	ACAACTTTGGTATCGTGGAAGG
GAPDH-Rv	GCCATCACGCCACAGTTTC

Supplementary Table 9 Genomic PCR primers and sequencing primers

Name	Sequence (5' to 3')
EGFP-Fw	GCCTCAGACAGTGGTTCAAAG
EGFP-Rv	TAACATATAGACAAACGCACACCG
EGFP-seq	TGAGCAAGGGCGAGGAG
B2M-Fw	GAGGGAAAGATACCAAGTCACGG
B2M-Rv	CATCAGTATCTCAGCAGGTGCCAC
B2M-seq	TGGAAATGGAATTGGGAG

Supplementary Table 10 Next generation sequencing primers

Name	Sequence (5' to 3')
Miseq EGFP-Fw	GATGTGTATAAGAGACAGGACCCTGAAGTTCATCTGCAC
Miseq EGFP-Rv	CGTGTGCTCTTCCGATCTGCTCCTGGACGTAGCCTT
Miseq ALK-Fw	GATGTGTATAAGAGACAGAAGAACTGGAAGCCCGA

Miseq ALK-Rv	CGTGTGCTCTTCCGATCTGTCAAACAGGCCACAATAATGAG
Miseq CACNA1D-Fw	GATGTGTATAAGAGACAGGACCCAGAGATACATGGCTATTT
Miseq CACNA1D-Rv	CGTGTGCTCTTCCGATCTAGGGCGGCCTCCC

Supplementary Table 11 Protein sequence of recombinant proteins

Protein coding sequence, Nuclear localization signal and His tag

Name	Sequence
**SpCas9**	**MDKKYSIGLDIGTNSVGWAVITDEYKVPSKKFKVLGNTDRHSIKKNLIGALLFDSGETAEA<br>TRLKRTARRRYTRRKNRICYLQEIFSNEMAKVDDSFFHRLEESFLVEEDKKHERHPIFGNI<br>VDEVAYHEKYPTIYHLRKKLVDSTDKADLRLIYLALAHMIKFRGHFLIEGDLNPDNSDVDK<br>LFIQLVQTYNQLFEENPINASGVDAKAILSARLSKSRRLENLIAQLPGEKKNGLFGNLIAL<br>SLGLTPNFKSNFDLAEDAKLQLSKDTYDDDLDNLLAQIGDQYADLFLAAKNLSDAILLSDI<br>LRVNTEITKAPLSASMIKRYDEHHQDLTLLKALVRQQLPEKYKEIFFDQSKNGYAGYIDG<br>GASQEEFYKFIKPILEKMDGTEELLVKLNREDLLRKQRTFDNGSIPHQIHLGELHAILRRQ<br>EDFYPFLKDNREKIEKILTFRIPYYVGPLARGNSRFAWMTRKSEETITPWNFEEVVDKGA<br>SAQSFIERMTNFDKNLPNEKVLPKHSLLYEYFTVYNELTKVKYVTEGMRKPAFLSGEQK<br>KAIVDLLFKTNRKVTVKQLKEDYFKKIECFDSVEISGVEDRFNASLGTYHDLLKIIKDKDFL<br>DNEENEDILEDIVLTLTLFEDREMIEERLKTYAHLFDDKVMKQLKRRRYTGWGRLSRKLIN<br>GIRDKQSGKTILDFLKSDGFANRNFMQLIHDDSLTFKEDIQKAQVSGQGDSLHEHIANLA<br>GSPAIKKGILQTVKVVDELVKVMGRHKPENIVIEMARENQTTQKGQKNSRERMKRIEEGI<br>KELGSQILKEHPVENTQLQNEKLYLYYLQNGRDMYVDQELDINRLSDYDVDHIVPQSFLK<br>DDSIDNKVLTRSDKNRGKSDNVPSEEVVKKMKNYWRQLLNAKLITQRKFDNLTKAERG<br>GLSELDKAGFIKRQLVETRQITKHVAQILDSRMNTKYDENDKLIREVKVITLKSKLVSDFR<br>KDFQFYKVREINNYHHAHDAYLNAVVGTALIKKYPKLESEFVYGDYKVYDVRKMIAKSEQ<br>EIGKATAKYFFYSNIMNFFKTEITLANGEIRKRPLIETNGETGEIVWDKGRDFATVRKVLSM<br>PQVNIVKKTEVQTGGFSKESILPKRNSDKLIARKKDWDPKKYGGFDSPTVAYSVLVVAKV<br>EKGKSKKLKSVKELLGITIMERSSFEKNPIDFLEAKGYKEVKKDLIIKLPKYSLFELENGRK<br>RMLASAGELQKGNELALPSKYVNFLYLASHYEKLKGSPEDNEQKQLFVEQHKHYLDEII<br>EQISEFSKRVILADANLDKVLSAYNKHRDKPIREQAENIIHLFTLTNLGAPAAFKYFDTTID<br>RKRYTSTKEVLDATLIHQSITGLYETRIDLSQLGGDGGGSGTRLPKKKRKVGGGSHHHH<br>HH Stop**
AsCas12a	MTQFEGFTNLYQVSKTLRFELIPQGKTLKHIQEQGFIEEDKARNDHYKELKPIIDRIYKTYAD<br>QCLQLVQLDWENLSAAIDSYRKEKTEETRNALIEEQATYRNAIHDYFIGRTDNLTDAINKRH<br>AEIYKGLFKAELFNGKVLKQLGTVTTTEHENALLRSFDKFTTYFSGFYENRKNVFSAEDIST<br>AIPHRIVQDNFPKFKENCHIFTRLITAVPSLREHFENVKKAIGIFVSTSIEEVFSFPFYNQLLT<br>QTQIDLYNQLLGGISREAGTEKIKGLNEVLNLAIQKNDETAHIIASLPHRFIPLFKQILSDRNTL<br>SFILEEFKSDEEVIQSFCKYKTLLRNENVLETAEALFNELNSIDLTHIFISHKKLETISSALCDH<br>WDTLRNALYERRISELTGKITKSAKEKVQRSLKHEDINLQEIISAAGKELSEAFKQKTSEILS<br>HAHAALDQPLPTTLKKQEEKEILKSQLDSLLGLYHLLDWFAVDESNEVDPEFSARLTGIKLE<br>MEPSLSFYNKARNYATKKPYSVEKFKLNFQMPTLASGWDVNKEKNNGAILFVKNGLYYLG

	IMPKQKGRYKALSFEPTEKTSEGFDKMYYDYFPDAAKMIPKCSTQLKAVTAHFQTHTTPIL<br>LSNNFIEPLEITKEIYDLNNPEKEPKKFQTAYAKKTGDQKGYREALCKWIDFTRDFLSKYTK<br>TTSIDLSSLRPSSQYKDLGEYYAELNPLLYHISFQRIAEKEIMDAVETGKLYLFQIYNKDFAK<br>GHHGKPNLHTLYWTGLFSPENLAKTSIKLNGQAELFYRPKSRMKRMAHRLGEKMLNKKLK<br>DQKTPIPDTLYQELYDYVNHRLSHDLSDEARALLPNVITKEVSHEIIKDRRFTSDKFFFHVPI<br>TLNYQAANSPSKFNQRVNAYLKEHPETPIIGIDRGERNLIYITVIDSTGKILEQRSLNTIQQFD<br>YQKKLDNREKERVAARQAWSVVGTIKDLKQGYLSQVIHEIVDLMIHYQAVVVLENLNFGFK<br>SKRTGIAEKAVYQQFEKMLIDKLNCLVLKDYPAEKVGGVLNPYQLTDQFTSFAKMGTQSG<br>FLFYVPAPYTSKIDPLTGFVDPFVWKTIKNHESRKHFLEGFDFLHYDVKTGDFILHFKMNRN<br>LSFQRGLPGFMPAWDIVFEKNETQFDAKGTPFIAGKRIVPVIENHRFTGRYRDLYPANELIA<br>LLEEKGIVFRDGSNILPKLLENDDSHAIDTMVALIRSVLQMRNSNAATGEDYINSPVRDLNG<br>VCFDSRFQNPEWPMDADANGAYHIALKGQLLLNHLKESKDLKLQNGISNQDWLAYIQELR<br>NPKKKRKVGGGSHHHHHH Stop

REFERENCES

1. Richardson, C.D., Ray, G.J., DeWitt, M.A., Curie, G.L. and Corn, J.E. (2016) Enhancing homology-directed genome editing by catalytically active and inactive CRISPR-Cas9 using asymmetric donor DNA. *Nat Biotechnol.*, **34**, 339-344.
2. Wang, Y., Liu, K.I., Sutrisnoh, N.B., Srinivasan, H., Zhang, J., Li, J., Zhang, F., Lalith, C.R.J., Xing, H., Shanmugam, R. *et al.* (2018) Systematic evaluation of CRISPR-Cas systems reveals design principles for genome editing in human cells. *Genome Biol.*, **19**, 62.
